# Toward Unified AI Drug Discovery with Multimodal Knowledge

**DOI:** 10.34133/hds.0113

**Published:** 2024-02-23

**Authors:** Yizhen Luo, Xing Yi Liu, Kai Yang, Kui Huang, Massimo Hong, Jiahuan Zhang, Yushuai Wu, Zaiqing Nie

**Affiliations:** ^1^ Institute for AI Industry Research (AIR), Tsinghua University, Beijing, China.; ^2^ Department of Computer Science and Technology, Tsinghua University, Beijing, China.; ^3^ School of Software and Microelectronics, Peking University, Beijing, China.; ^4^ Beijing Academy of Artificial Intelligence (BAAI), Beijing, China.

## Abstract

**Background:** In real-world drug discovery, human experts typically grasp molecular knowledge of drugs and proteins from multimodal sources including molecular structures, structured knowledge from knowledge bases, and unstructured knowledge from biomedical literature. Existing multimodal approaches in AI drug discovery integrate either structured or unstructured knowledge independently, which compromises the holistic understanding of biomolecules. Besides, they fail to address the missing modality problem, where multimodal information is missing for novel drugs and proteins. **Methods:** In this work, we present KEDD, a unified, end-to-end deep learning framework that jointly incorporates both structured and unstructured knowledge for vast AI drug discovery tasks. The framework first incorporates independent representation learning models to extract the underlying characteristics from each modality. Then, it applies a feature fusion technique to calculate the prediction results. To mitigate the missing modality problem, we leverage sparse attention and a modality masking technique to reconstruct the missing features based on top relevant molecules. **Results:** Benefiting from structured and unstructured knowledge, our framework achieves a deeper understanding of biomolecules. KEDD outperforms state-of-the-art models by an average of 5.2% on drug–target interaction prediction, 2.6% on drug property prediction, 1.2% on drug–drug interaction prediction, and 4.1% on protein–protein interaction prediction. Through qualitative analysis, we reveal KEDD’s promising potential in assisting real-world applications. **Conclusions:** By incorporating biomolecular expertise from multimodal knowledge, KEDD bears promise in accelerating drug discovery.

## Introduction

Drug discovery aims to design novel therapeutic agents that respond to a certain disease and reduce their potential side effects on patients [1–3]. The understanding of biomolecules, which entails either drugs or proteins, builds the foundation of drug discovery processes [4]. Such molecular expertise usually resides within three different modalities: **molecular structures like SMILES strings of molecules and amino acid sequences of proteins** [5], **structured knowledge from knowledge graphs** [6], and **unstructured knowledge from biomedical documents** [7]. These modalities complement each other, providing a holistic view to guide researchers in pharmaceutical applications.

While artificial intelligence (AI) models that mine intrinsic patterns from molecular structures and protein sequences [8–11] have achieved great success in assisting drug discovery, recent advances of multimodal models have shown the benefits of incorporating structured and unstructured knowledge in numerous downstream tasks, including drug–target interaction prediction (DTI) [12–14], drug–drug interaction prediction (DDI) [15–17], and protein–protein interaction prediction (PPI) [18,19]. However, existing models are mostly restricted to a single task, and none of them attempt to take advantage of both structured and unstructured knowledge. This limits not only the application scope but also the capability of AI systems to holistically understand the intrinsic properties and functions of biomolecules. Besides, multimodal knowledge is occasionally unavailable for newly discovered drugs and proteins due to the extensive cost of manual annotations. This formidable challenge, known as the missing modality problem [20–22], hampers the capability of multimodal deep learning models in assisting real-world drug development.

In this work, we propose KEDD, a unified end-to-end deep learning framework for **K**nowledge-**E**mpowered **D**rug **D**iscovery to solve the aforementioned problems. KEDD simultaneously harvests biomedical expertise from molecular structures, structured knowledge from knowledge graphs, and unstructured knowledge from biomedical literature. KEDD could be flexibly applied to a wide range of AI drug discovery tasks. The framework first incorporates independent off-the-shelf representation learning models to extract dense features from each modality. Then, it performs feature fusion by concatenating the multimodal features and calculates the results with a prediction network. To alleviate the missing modality problem for structured knowledge, KEDD leverages multihead sparse attention to reconstruct features based on the most relevant biomolecules, and proposes a modality masking technique to improve the training of sparse attention.

Comprehensive experiments on 13 popular benchmarks demonstrate KEDD’s capability in solving wide downstream tasks in AI drug discovery. KEDD outperforms state-of-the-art models by an average of 5.2% on DTI, 2.6% on drug property prediction (DP), 1.2% on DDI, and 4.1% on PPI. Additionally, qualitative results shed light on KEDD’s joint comprehension of different modalities and its potential in assisting real-world applications.

Our main contributions are summarized as follows:

• We present KEDD, a unified, end-to-end framework incorporating multimodal knowledge of molecular structure, structured knowledge within knowledge graphs, and unstructured knowledge within biomedical documents for drug discovery.

• We propose sparse attention and modality masking to alleviate the missing modality problem for knowledge graphs.

• We demonstrate the state-of-the-art performance of KEDD in wide-ranging AI drug discovery tasks.

## Methods

In this section, we start with a brief introduction of preliminaries and denotations, followed by a introduction of the overall architecture of KEDD. Then, we detail two strategies to incorporate structured and unstructured knowledge, including direct searching and reconstruction via sparse attention. Finally, we present the implementation details of KEDD on several downstream benchmarks.

### Preliminaries

KEDD focuses on two types of biomolecules involved in drug discovery: drugs and proteins. Each component further consists of information from three modalities, namely, molecular structure, structured knowledge, and unstructured knowledge. Formally:

$$\begin{array}{c} d=(D_{S},D_{SK},D_{UK})\in D\\ p=(P_{S},P_{SK},P_{UK})\in P \end{array}$$ (1)

where *d* refers to a drug, *p* refers to a protein, and *D*, *P* refers to the drug and protein spaces. The drug structure *D*_S_ is profiled as a two-dimensional (2D) molecular graph (*V*, *E*), where *V* denotes atoms and *E* denotes molecular bonds. The protein structure *P*_S_ is profiled as a sequence [*p*_1_, *p*_2_, …, *p_M_*] of length *M*, where *p_i_* corresponds to an amino acid. The knowledge base is formulated as *KB* = (*E*, *R*), where *E* is the entity set and *R* is composed of numerous triplets (*h*, *r*, *t*). *h*, *t* ∈ *E* are the head and tail entity, respectively, and *r* is the relation type. The structured knowledge *D*_SK_ ∈ *E* or *P*_SK_ ∈ *E* is formulated as the corresponding entity in the knowledge base. The unstructured knowledge *D*_UK_ or *P*_UK_ is formulated as a text sequence [*t*_1_, *t*_2_, ⋯, *t_L_*] of length *L*.

AI drug discovery tasks aim to uncover the properties of novel drugs and proteins, as well as the interactions between them. They can be formulated as learning mapping functions from the drug, protein, or joint spaces to binary values. Formally:

**•** DTI predicts whether a given drug binds to a specific protein target. This task sheds light on improving the effectiveness of drugs and reducing their toxicity to the human body [23]. The task is formulated as learning *F*_DTI_ : *D* × *P* → {0, 1}.

**•** DP predicts the existence of biomolecular properties such as toxicity, permeability, and side effects. The task is formulated as learning *F*_DP_ : *D* → {0, 1}.

**•** DDI predicts whether two drugs interact with each other, which plays an important role in co-administration. The task is formulated as learning *F*_DDI_ : *D* × *D* → {0, 1}.

**•** PPI aims at predicting different types of interaction relationships between proteins mainly based on their amino acid sequences. The task is beneficial to applications such as identifying the functions and drug abilities of biomolecules [24]. The task is formulated as learning *F*_PPI_ : *P* × *P* → {0, 1}*^n^*, where *n* is the number of relation types.

For DTI, DDI, and PPI, the binary output signifies the presence of a particular category of interaction between the provided drugs or proteins. For DP, the binary output indicates if the molecule holds a specific property. Due to their similar formulations, we endeavor to build a unified end-to-end deep learning framework to solve these tasks with minimal modifications.

### KEDD architecture

**Fig. 1.**
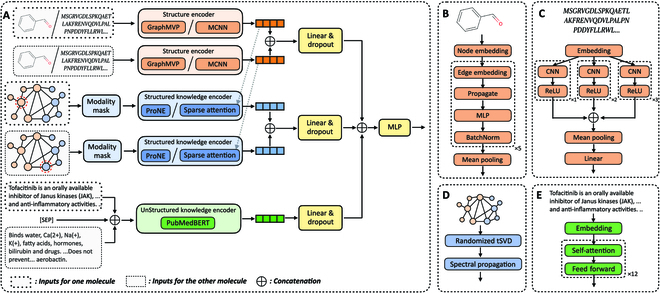
The KEDD architecture. (A) The overall feature fusion framework. Inputs for molecules can be a drug, a protein, or empty depending on the downstream task. (B) Network architecture of the drug structure encoder GraphMVP. (C) Network architecture of the protein structure encoder MCNN. (D) Workflow of the structured knowledge encoder ProNE. (E) Network architecture of the unstructured knowledge encoder PubMedBERT.

Figure 1 illustrates the overall KEDD architecture. In the following section, we detail each component of KEDD.

#### Drug structure encoder

To encode the molecular graph *D*_S_ = (*V*, *E*), we use GraphMVP [8], a five-layer GIN [25] pretrained on both 2D molecular graphs and 3D molecular genomics. As illustrated in Fig. 1B, GraphMVP first calculates the initial node embedding matrix ${\tilde{X}}^{\left(0\right)}\in {\mathbb{R}}^{|V|\times 120}$ based on the type and chirality for each atom. Then, each layer of GIN propagates the node features from the previous layer in a message-passing manner. Specifically, at the *k*th layer, it first calculates the edge embedding matrix ${\tilde{E}}^{\left(k\right)}\in {\mathbb{R}}^{|E|\times 6}$ based on the bond type and bond direction. Then, the node features are updated as follows:

$${\tilde{X}}_{v}^{\left(k\right)}={\text{MLP}}^{\left(k\right)}\left({\tilde{X}}_{v}^{\left(k-1\right)}+\sum_{\left(u,v\right)\in E}\;\left({\tilde{X}}_{u}^{\left(k-1\right)}+{\tilde{E}}_{j}^{\left(k\right)}\right)\right),v\in V,$$ (2)

where *j* denotes the corresponding edge connecting *u* and *v*, and *MLP*^(*k*)^ is a trainable network composed of a fully connected layer, a ReLU activation, and another fully connected layer. The structure feature $z_{S}^{D}$ is calculated by mean pooling over the node features of the last layer:

$$z_{S}^{D}=\frac{1}{|V|}\sum_{v\in V}\;{\tilde{X}}_{v}^{\left(5\right)}.$$ (3)

#### Protein structure encoder

To encode protein structure *P_S_* = [*p*_1_, *p*_2_, ⋯, *p_m_*], we use multiscale convolutional neural network (MCNN) [26], a network with three branches of stacked convolutional layers. The MCNN architecture is shown in Fig. 1C. It first incorporates an embedding layer to transform *P_S_* into an embedding matrix $\tilde{P}\in {\mathbb{R}}^{m\times 128}$. Then, it passes $\tilde{P}$ to each branch, which composes one, two, and three convolution layers with a kernel size of 3 × 3, followed by ReLU activation after each convolution layer. Finally, it applies max pooling over the sequence, concatenates the outputs from each branch, and feeds the concatenation results into a fully connected layer. Formally, the structural feature of a protein is calculated as follows:

$$z_{S}^{P}=W^{P}\left(MF_{1}\left(\tilde{P}\right)⊕F_{2}\left(\tilde{P}\right)⊕F_{3}\left(\tilde{P}\right)\right),$$ (4)

where *F*_1_, *F*_2_, *F*_3_ are three branches of stacked convolution layers followed by ReLU activation, ⊕ denotes concatenation, *M*(⋅) denotes max pooling, and *W^P^* ∈ ℝ^384 × 128^ is a trainable matrix.

#### Structured knowledge encoder

To encode the structured knowledge *D_SK_* and *P_SK_*, we leverage ProNE [27], a fast and efficient network embedding algorithm, which is illustrated in Fig. 1D. ProNE transforms the knowledge graph *KB* into an embedding matrix ${\tilde{H}}^{\left(\mathrm{KB}\right)}={\mathbb{R}}^{|E|\times 256}$ through sparse randomized truncated singular value decomposition (tSVD) decomposition and spectral propagation enhancement. The structured knowledge features are obtained as follows:

$$z_{\mathrm{SK}}^{D}={\tilde{H}}_{D_{\mathrm{SK}}}^{\left(\mathrm{KB}\right)},z_{\mathrm{SK}}^{P}={\tilde{H}}_{P_{\mathrm{SK}}}^{\left(\mathrm{KB}\right)}$$ (5)

#### Unstructured knowledge encoder

To encode the unstructured knowledge *D_UK_* and *P_UK_*, we adopt PubMedBERT [28], a language model pretrained on biomedical corpus. As illustrated in Fig. 1E, PubMedBERT is composed of 12 Transformer layers, each composing a self-attention module and a feed-forward network. Given the input tokens [*t*_1_, *t*_2_, ⋯, *t_L_*] where *t*_1_=[CLS], PubMedBERT transforms it into a series of contextualized embeddings [*h*_1_, *h*_2_, ⋯, *h_L_*], where *h_i_* ∈ ℝ^768^. Features for unstructured knowledge *z_UK_* are calculated by feeding the [CLS] embedding into a fully connected layer with dropout:

$$z_{\mathrm{UK}}=F_{\mathrm{UK}}\left(\left[t_{1},t_{2},\cdots ,t_{L}\right]\right)=\text{Dropout}\;\left(W^{\mathrm{UK}}h_{1}+b^{\mathrm{UK}}\right),$$ (6)

where *W^UK^* ∈ ℝ^768×*d_U K_*^, *b^U K^* ∈ ℝ*^d_U K_^* are trainable parameters.

#### Multimodal feature fusion

The feature vectors with respect to each modality for tasks defined in the “Preliminaries” section are detailed as follows.

For DTI:

$$\begin{array}{c} \begin{array}{cc} & \;z_{S}=\text{Dropout}\left(W^{S}\left(z_{S}^{D}⊕z_{S}^{P}\right)+b^{S}\right),\\ & \;z_{\mathrm{SK}}=\text{Dropout}\left(W^{\mathrm{SK}}\left(z_{\mathrm{SK}}^{D}⊕z_{\mathrm{SK}}^{P}\right)+b_{\mathrm{SK}}\right),\\ & z_{\mathrm{UK}}=F_{\mathrm{UK}}\left(D_{\mathrm{UK}}⊕\left[\mathrm{SEP}\right]⊕P_{\mathrm{UK}}\right). \end{array} \end{array}$$ (7)

For DP:

$$\begin{array}{l} \;z_{S}=\text{Dropout}\left(W^{S}z_{S}^{D}+b_{S}\right),\\ \;z_{\mathrm{SK}}=\text{Dropout}\left(W^{\mathrm{SK}}z_{\mathrm{SK}}^{D}+b_{\mathrm{SK}}\right),\\ z_{\mathrm{UK}}=F_{\mathrm{UK}}\left(D_{\mathrm{UK}}\right). \end{array}$$ (8)

For DDI:

$$\begin{array}{c} \;z_{S}=\text{Dropout}\left(W^{S}\left(z_{S}^{D_{1}}⊕z_{S}^{D_{2}}\right)+b^{S}\right),\\ \;z_{\mathrm{SK}}=\text{Dropout}\left(W^{\mathrm{SK}}\left(z_{\mathrm{SK}}^{D_{1}}⊕z_{\mathrm{SK}}^{D_{2}}\right)+b_{\mathrm{SK}}\right),\\ z_{\mathrm{UK}}=F_{\mathrm{UK}}\left(D_{1,U\;K}⊕\left[\mathrm{SEP}\right]⊕D_{2,U\;K}\right), \end{array}$$ (9)

where *D*_1_, *D*_2_ denote two input drugs.

For PPI:

$$\begin{array}{c} \;z_{S}=\text{Dropout}\left(W^{S}\left(z_{S}^{P_{1}}⊕z_{S}^{P_{2}}\right)+b^{S}\right),\\ \;z_{\mathrm{SK}}=\text{Dropout}\left(W^{\mathrm{SK}}\left(z_{\mathrm{SK}}^{P_{1}}⊕z_{\mathrm{SK}}^{P_{2}}\right)+b_{\mathrm{SK}}\right),\\ z_{\mathrm{UK}}=F_{\mathrm{UK}}\left(P_{1,U\;K}⊕\left[\mathrm{SEP}\right]⊕P_{2,U\;K}\right), \end{array}$$ (10)

where *P*_1_, *P*_2_ denote two input proteins.

*W^S^*, *W^SK^*, *b^S^*, *b^SK^* are trainable parameters. Notably, in DTI, DDI, and PPI, the textual descriptions of two biomolecules are concatenated with a [SEP] token before feeding them into PubMedBERT. Such a design enables the language model to better capture the co-occurrence of key information, thus supporting interaction prediction.

Finally, the features from molecular structures, structured knowledge, and unstructured knowledge are concatenated and passed into a multilayer perceptron to generate prediction results. We incorporate cross-entropy loss as the objective function:

$$\begin{array}{c} \hat{y}=\text{MLP}\left(z_{S}⊕z_{\mathrm{SK}}⊕z_{U\;K}\right),\\ L=-\left[y\text{log}\hat{y}+\left(1-y\right)\text{log}\left(1-\hat{y}\right)\right], \end{array}$$ (11)

where *y* ∈ {0, 1} is the ground-truth label.

### Multimodal knowledge acquisition

The majority of existing datasets for AI drug discovery only provide structural information *D_S_*, *P_S_* for drugs and proteins. As shown in the “Methods” section, we propose two strategies to obtain the multimodal knowledge *D_SK_*, *D_UK_*, *P_SK_*, *P_U K_*, i.e., direct acquisition and sparse attention-based reconstruction.

#### Direct acquisition from the BMKG dataset

Based on public repositories [29–33], we build BMKG, a dataset containing molecular structure, interacting relationships, and expert-written textual descriptions for 6,917 drugs and 19,992 proteins. In total, BMKG contains 2,223,850 drug–drug links, 47,530 drug–protein links, and 633,696 protein–protein links. Details of our construction process are presented in Supplementary Section A and Fig. S1. The BMKG dataset functions as a dictionary, wherein biomolecular structures serve as keys, while structured and unstructured knowledge constitute values. We can efficiently acquire multimodal knowledge for drugs and proteins by conducting searches within BMKG based on identical SMILES strings or amino acid sequences.

#### Mitigating missing modality with sparse attention and modality masking

Ideally, each molecule is accompanied by the corresponding structured and unstructured knowledge. However, as elucidated in Table 1, a considerable proportion of molecules, especially those recently discovered, remain unaccounted for in existing databases owing to the substantial expenses associated with manual annotation processes. This formidable missing modality problem significantly compromises the application of multimodal AI drug discovery approaches in real-world scenarios.

**Table 1.** A summary of benchmark datasets. The total number of molecules in the dataset is to the right of /, and the number of molecules linked to BMKG is to the left of /.

Task	Dataset	# Drugs	# Proteins	# Samples
DTI	BMKG-DTI	2,803/2,803	2,810/2,810	47,391
	Yamanishi08	488/791	944/989	10,254
DP	BBBP	841/2,039	-	2,039
	ClinTox	556/1,478	-	1,478
	Tox21	2,191/7,831	-	7,831
	SIDER	677/1,427	-	1,427
	ToxCast	1,875/8,575	-	8,575
	MUV	193/93,087	-	93,087
	HIV	297/41,127	-	41,127
	BACE	312/1,513	-	1,513
DDI	Luo’s	657/721	-	494,551
PPI	SHS27k	-	1,632/1,690	7,624
	SHS148k	-	4,943/5,189	44,488

**Fig. 2.**
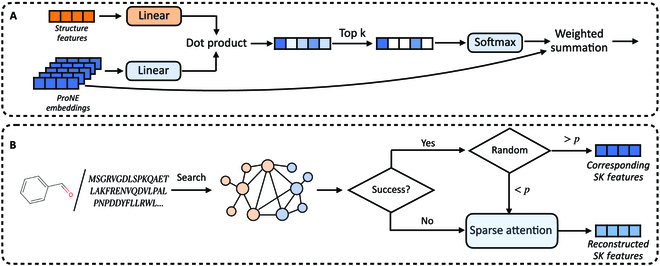
The multimodal knowledge acquisition pipeline. (A) Sparse attention pipeline that takes the structural features as queries to obtain top-*k* relevant entities within BMKG. (B) We search for identical biomolecular structures in BMKG to obtain multimodal knowledge. If the search fails or the modality masking is triggered, we apply sparse attention to reconstruct the structured knowledge features.

To mitigate this issue, we leverage sparse attention [34] shown in Fig. 2A to reconstruct the structured knowledge features $z_{\mathrm{SK}}^{D}$ and $z_{\mathrm{SK}}^{P}$ by querying the most relevant entities within the knowledge graph based on molecular structure. We project the molecular structure features $z_{S}^{D}$ or $z_{S}^{P}$ to the feature space of structured knowledge with a fully connected layer. We use the projected results ${\tilde{z}}_{S}^{D}$ or ${\tilde{z}}_{S}^{P}$ as queries, and the knowledge graph embedding matrix ${\tilde{H}}^{\left(\mathrm{KB}\right)}$ calculated in the “Results” section as keys and values. The structured knowledge features ${\tilde{z}}_{\mathrm{SK}}^{D}$ or ${\tilde{z}}_{\mathrm{SK}}^{P}$ are reconstructed as follows:

$$\begin{array}{c} Q=W^{Q}{\tilde{z}}_{S}^{D/P},\;K=W^{K}{\tilde{H}}^{\left(\mathrm{KB}\right)},\;V=W^{V}{\tilde{H}}^{\left(K\;B\right)}\\ A=\frac{{\mathrm{QK}}^{T}}{\sqrt{d_{\mathrm{SK}}}},\;\tilde{A}=\text{SoftMax}\left(\mathrm{Top}\left(A,k\right)\right)\\ \;{\tilde{z}}_{\mathrm{SK}}^{D/P}=\tilde{A}V \end{array}$$ (12)

where *W_Q_*, *W_K_* ∈ ℝ^*d_SK_* × *d_SK_*^ are trainable parameters. Top(*A*, *k*) identifies the *k* largest elements within *A* and withdraws the remaining elements by assigning a similarity score of −∞. Different from traditional attention-based networks, *W_V_* is fixed as an identity matrix. In this way, the sparse attention can be viewed as a trainable interpolation module that dynamically explores and allocates different weights to the most relevant *k* entities within the knowledge graph.

On occasions where the missing modality problem is not too severe, the number of molecules and proteins that require reconstruction could be insufficient to train the sparse attention module. As depicted in Fig. 2B, we propose a modality masking strategy to address this issue. With a probability of *P*, we mask the structured knowledge inputs *D_SK_* and *P_SK_* obtained by direct acquisition, and activate the reconstruction process with sparse attention. This strategy creates additional training samples proportional to the original training set for the sparse attention module. Notably, constituting ${\tilde{z}}_{\mathrm{SK}}^{D}$ and ${\tilde{z}}_{\mathrm{SK}}^{P}$ with the reconstruction features ${\tilde{z}}_{\mathrm{SK}}^{D}$ and ${\tilde{z}}_{\mathrm{SK}}^{P}$ can be perceived as a form of data augmentation, thereby enhancing the robustness of our framework.

### Evaluation

KEDD is applied on four popular downstream tasks with 13 benchmark datasets summarized in Table 1.

**•** DTI. We adopt two binary classification datasets: Yamanishi08 [35] and BMKG-DTI. Yamanishi08 is collected mainly from the KEGG database [31]. BMKG-DTI is constructed based on BMKG. More details of this dataset are available in Supplementary Section C and Fig. S2. We perform 5-fold cross-validation for the warm-start, cold-drug, and cold-protein settings, and 9-fold cross-validation for the cold-cluster setting, similar to [36]. Under the warm-start setting, drugs and proteins are randomly partitioned. Under the cold-drug, cold-protein, and cold-cluster settings, drugs, proteins, and both in the test set, respectively, are unseen during training. We report the area under the receiver operating characteristic curve (AUROC) and the area under the precision–recall curve (AUPR) as evaluation metrics.

**•** DP. We select eight representative binary classification datasets from MoleculeNet [37], a widely adopted benchmark for molecular machine learning. We adopt Scaffold split where drugs within the test set are distinct to those in the training set. The train–validation–test ratio is 8:1:1. We report AUROC for this task.

**•** DDI. We adopt Luo’s dataset [38], randomly split the binary classification dataset with a train–validation–test ratio of 8:1:1, and report AUROC and AUPR.

**•** PPI. We leverage the revised version of multilabel classification datasets SHS27k and SHS148k [39]. We follow the breadth first search (BFS) and depth first search (DFS) strategy [18] to split the dataset with a train–test ratio of approximately 4:1. We adopt the Micro F1 score as the evaluation metric.

Details of evaluation datasets and splitting protocols are presented in Supplementary Section B.

### Implementation details

Across our experiments, we set the number of attention heads within sparse attention as 4, and the number of extracted entities *k* as 16. The modality masking probability *P* is set with 0.05 during training and 0 during testing. To avoid information leakage, we remove connections between drugs and proteins in the test set of DDI, DTI, and PPI datasets from BMKG before calculating knowledge graph embeddings. KEDD adopts the Adam optimizer [40] with a weight decay of 10^−6^ to update model parameters. The KEDD model is trained on a single A100 GPU with 40 GB memory, with a maximum training cost of 1 day. Each experiment is performed three times with different seeds. The hyperparameters for each dataset are adapted by randomized grid search, and their choices are shown in Table S1.

## Results

### Performance evaluation on downstream tasks

In this section, we present and analyze the results of KEDD and baseline models on four downstream tasks. We demonstrate that structured and unstructured knowledge could provide valuable biomedical insights for drug discovery, and KEDD attains a comprehensive understanding of biomolecules with multimodal data. A detailed introduction of baselines is presented in Supplementary Section E.

#### Performance evaluation on DTI

**Fig. 3.**
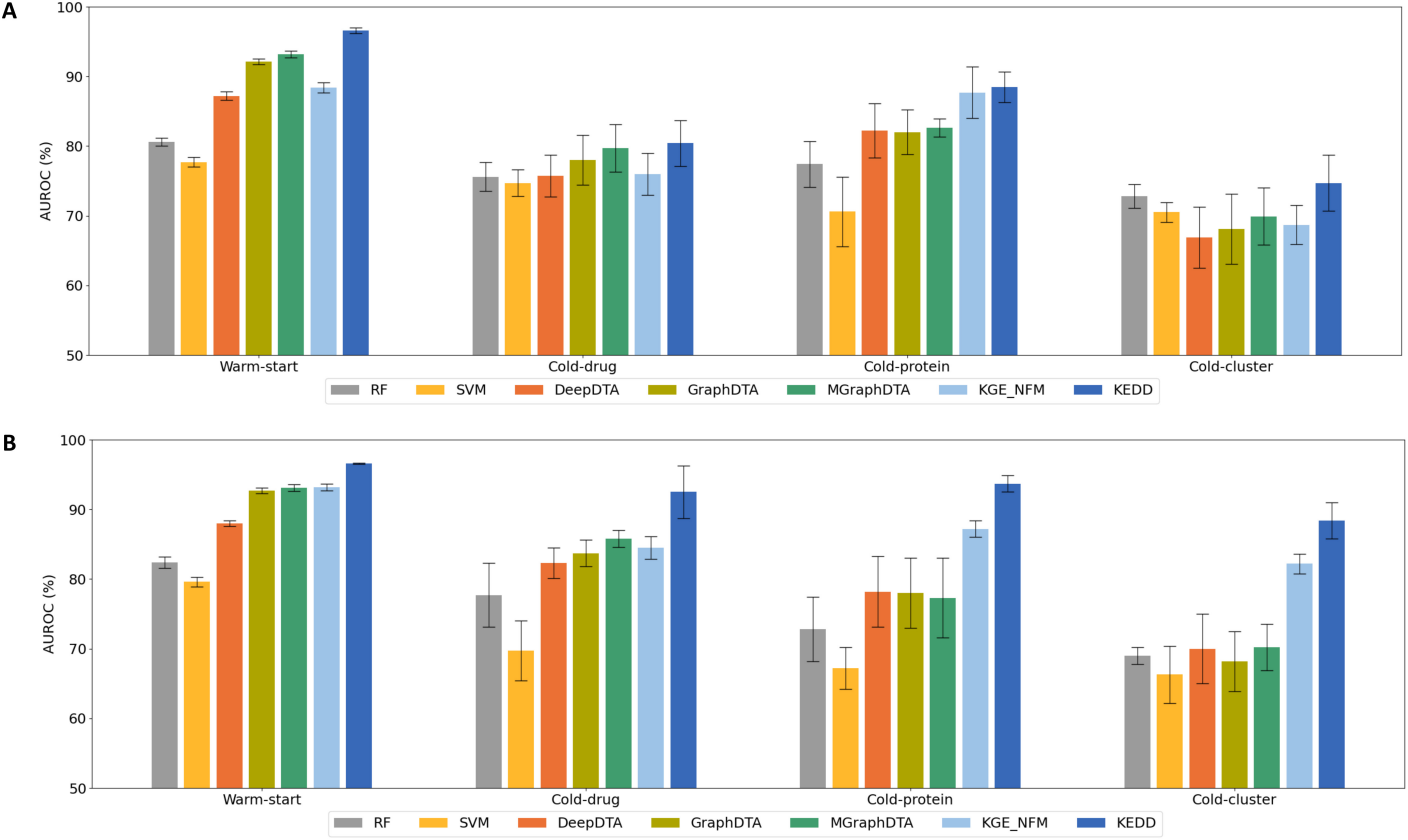
Performance comparison for drug–target interaction prediction under warm-start and cold-start settings. (A) AUROC on Yamanishi08 dataset. (B) AUROC on BMKG dataset.

We compare KEDD against machine learning models RF [41] and SVM [42], unimodal baselines including DeepDTA [43], GraphDTA [44], and MGraphDTA [26], and the multimodal approach KGE_NFM [13]. The AUROC results are shown in Fig. 3. The complete experimental results are displayed in Tables S2 and S3.

Under the warm-start setting, deep learning models surpass machine learning baselines by a remarkable margin. Besides, models such as GraphDTA and MGraphDTA that incorporate graph neural network (GNN)-based drug encoders significantly outperform models like DeepDTA that incorporate CNN-based drug encoders, which corroborates prior studies [44]. While KGE_NFM incorporates simple molecular fingerprints to model molecular structure, it also yields promising results, because of the incorporation of knowledge graph embeddings. Remarkably, KEDD achieves the best results on both datasets. Compared to the state-of-the-art model MGraphDTA, KEDD achieves a notable gain of 3.4% and 3.5% in AUROC under the warm-start setting on Yamanishi08 and BMKG-DTI (paired *t* test, all *P* <1.3 × 10^−6^).

In comparison with the overoptimistic results under the warm-start setting, the performance of AI models declines significantly under cold-start settings. Under the cold-cluster setting that is the most challenging, deep learning baselines even underperform RF on Yamanishi08 dataset. Compared to structure-based models, multimodal approaches such as KEDD and KGE_NFM mitigate the cold-start problem and achieve superior performance. On Yamanishi08, KEDD achieves state-of-the-art results under the cold-drug and cold-cluster settings (paired *t* tests, all *P* < 1.0 × 10^−2^) and shows minor statistical difference with KGE_NFM (paired *t* test, *P* > 5.0 × 10^−2^) under the cold-protein setting. Notably, on BMKG-DTI where the missing modality problem does not exist, KEDD exhibits profound improvements over existing models with an average performance gain of 8.1%, 7.5%, and 5.2% on cold-drug, cold-protein, and cold-cluster scenarios, respectively (paired *t* tests, all *P* < 2.9 × 10^−3^). It even achieves competitive results with that of the warm-start setting. These results demonstrate the benefits of incorporating structured and unstructured knowledge, especially for molecules that are out of the generalization scope of structure-based models.

#### Performance evaluation on DP

Comparisons between KEDD and machine learning models including RF and SVM as well as unimodal baselines including MolCLR [9], KV-PLM [10], MoMu [45], MoCL [46], and GraphMVP [8] are presented in Table 2. KEDD achieves significant performance gains across four of eight benchmarks including BBBP, ClinTox, Tox21, and ToxCast (paired *t* tests, all *P* < 4.1 × 10^−2^). These datasets encompass a relatively limited number of training samples, and the integration of multimodal knowledge endows KEDD with a more comprehensive understanding of the constrained data available. On the other two small datasets SIDER and BACE, KEDD yields an improvement of 2.3% and 2.1%, respectively, over the unimodal counterpart GraphMVP (paired *t* tests, all *P* < 1.6 × 10^−2^). However, it shows minor performance gain with RF on SIDER and underperforms the deep learning model SVM on BACE. We attribute this to the Scaffold split, which makes it challenging for deep learning models to grasp transferable characteristics based on a few thousands of training samples. On MUV and HIV, KEDD shows little statistical difference to GraphMVP (paired *t* tests, all *P* >3.3 × 10^−1^). We speculate that molecules within these two datasets are mostly under investigation and distinct from those recorded in BMKG, which results in the deterioration of KEDD to a unimodal paradigm. On average, KEDD yields an improvement of 2.6% in AUROC (paired *t* test, *P* < 1.3 × 10^−2^) over the state-of-the-art model GraphMVP. The promising outcomes validate the efficacy of integrating multimodal knowledge in DP, an aspect that has been disregarded in prior studies.

**Table 2.** Performance comparison in AUROC (%) for drug property (DP) prediction on MoleculeNet. The best results are marked in bold, and the second-best results are underlined.

Model	BBBP	ClinTox	SIDER	Tox21	ToxCast	MUV	HIV	BACE	Average
RF	64.9_±1.7_	66.2_±1.0_	65.8_±2.0_	69.7_±1.1_	59.3_±1.2_	69.0_±2.5_	73.3_±0.5_	79.0_±0.9_	68.4
SVM	64.5_±0.0_	70.6_±0.1_	56.8_±0.0_	65.1_±0.1_	58.2_±0.0_	64.1_±0.1_	69.2_±0.0_	**86.5**_±0.0_	66.9
GIN	65.4_±2.4_	74.9_±0.8_	61.6_±1.2_	58.0_±2.4_	58.8_±5.5_	71.0_±2.5_	75.3_±0.5_	72.6_±4.9_	67.2
MolCLR	71.1_±1.4_	61.1_±3.6_	57.7_±2.0_	74.0_±1.0_	61.6_±0.6_	73.2_±2.1_	74.4_±1.3_	76.7_±3.0_	68.7
KV-PLM	66.9_±1.1_	84.3_±1.5_	55.3_±0.9_	64.7_±1.8_	58.6_±0.4_	60.2_±2.9_	68.8_±4.5_	71.5_±2.1_	66.3
MoMu	70.5_±2.0_	79.9_±4.1_	60.5_±0.9_	75.6_±03_	63.4_±0.5_	70.5_±1.4_	75.9_±0.8_	76.7_±2.1_	71.6
MoCL	71.4_±1.1_	81.4_±1.0_	61.9_±0.4_	72.5_±1.0_	62.6_±0.5_	72.3_±0.3_	74.7_±0.6_	79.9_±0.4_	72.1
GraphMVP	72.4_±1.6_	77.5_±4.2_	63.9_±1.2_	74.4_±0.5_	63.1_±0.4_	**75.0**_±1.0_	77.0_±1.0_	81.2_±0.9_	73.1
KEDD (w/o SK)	72.7_±1.0_	86.2_±2.9_	61.9_±0.8_	74.9_±0.5_	63.3_±0.5_	72.0_±1.1_	76.2_±1.7_	81.3_±2.0_	73.6
KEDD (w/o UK)	72.2_±1.2_	72.5_±6.4_	63.9_±0.6_	75.8_±0.3_	62.8_±1.2_	71.2_±0.7_	75.3_±0.8_	82.5_±1.2_	72.0
KEDD (w/o SA)	72.3_±1.1_	87.2_±1.3_	62.8_±1.5_	75.1_±1.0_	63.9_±0.2_	72.8_±0.6_	76.3_±0.9_	82.4_±0.4_	74.1
KEDD	**73.6** **_±1.1_**	**88.4** **_±0.7_**	**66.0** **_±1.4_**	**76.8** **_±0.4_**	**64.9** **_±0.5_**	74.7_±0.4_	**77.3**_±0.3_	83.5_±0.3_	**75.7**

w/o SK, without structured knowledge; w/o UK, without unstructured knowledge; w/o SA, without sparse attention.

#### Performance evaluation on DDI

For this task, we adopt machine learning baselines including RF and SVM, unimodal baselines including DeepDTnet [47], DTINet [38], DeepR2cov [48], and MSSL2drug [49], and multimodal baselines including DDIMDL [50] and KGE_NFM [13]. The experimental results are shown in Table 3. Both machine learning baselines and deep learning baselines achieve promising results, indicating that both molecular structures and network topology provide valuable clues for identifying drug–drug interactions. Notably, KEDD achieves state-of-the-art results on Luo’s dataset in AUROC (paired *t* test, *P* < 2.1 × 10^−13^). While the AUPR score of KEDD is on par with MSSL2drug, our model exhibits significantly better stability between different runs. These results highlight the significance of jointly reasoning over molecular structures, knowledge graphs, and biomedical texts in this task.

**Table 3.** Performance comparison in AUROC (%) and AUPR (%) for drug–drug interaction prediction (DDI) on Luo’s dataset

Model	AUROC (%)	AUPR (%)
RF	82.1_±0.6_	80.7_±1.0_
SVM	79.7_±1.1_	79.3_±1.4_
DeepDTnet^a^	92.3_±0.8_	92.1_±1.0_
DTINet^a^	92.9_±0.6_	92.7_±0.9_
DeepR2cov^a^	93.1_±0.9_	91.2_±1.2_
MSSL2drug^a^	95.1_±0.4_	**94.4**_**±1.1**_
KGE_NFM^a^	91.6_±0.8_	90.7_±1.0_
DDIMDL^a^	91.3_±0.9_	90.5_±1.4_
KEDD (w/o SK)	96.3_±0.1_	91.7_±0.2_
KEDD (w/o UK)	97.1_±0.1_	92.9_±0.2_
KEDD (w/o SA)	97.4_±0.1_	94.1_±0.2_
KEDD	**97.5**_**±0.1**_	**94.4**_**±0.2**_

^a^ These results are taken from MSSL2drug [49].

#### Performance evaluation on PPI

In Table 4, we show the results of KEDD on the SHS27k and SHS148k dataset, compared against machine learning baselines including RF and SVM, unimodal baselines including PIPR [39] and ESM-650M [11], as well as multimodal baselines including GNN-PPI [18] and OntoProtein [19]. On SHS27k, KEDD outperforms baselines under the DFS setting by 2.7% to 10.8% (paired *t* tests, all *P* < 3.3 × 10^−2^). Under the BFS setting that is more challenging, KEDD outperforms multimodal baselines that consist of a similar amount of parameters, but shows minor statistical difference with ESM-650M (paired *t* test, *P* > 4.2 × 10^−1^), the scale of which exceeds KEDD by an order of magnitude (650M versus 115M). On SHS148k, KEDD achieves overwhelming advantages, outperforming ESM-650M by 6.2% and 2.1% absolute gains on the BFS and DFS settings, respectively (paired *t* test, *P* < 1.8 × 10^−2^). We speculate that the disparity between the two datasets lies in scale, with the number of proteins within SHS27k being inadequate for training our model from scratch. In comparison, ESM-650M has attained a good grasp of protein sequences by pretraining with billions of proteins, probably including those within the test set of our datasets. While KEDD opts for MCNN due to computational constraints, we expect a better performance by leveraging more powerful protein sequence encoders. 

**Table 4.** Performance comparison in Micro F1 (%) for protein–protein interaction prediction (PPI) on SHS27k and SHS148k datasets. The best results are marked in bold, and the second-best results are underlined.

Model	SHS27k		SHS148k	
	DFS	BFS	DFS	BFS
RF	35.6_±2.2_	37.7_±1.6_	43.3_±3.4_	39.0_±1.9_
SVM	53.1_±5.2_	43.0_±6.0_	58.6_±0.1_	49.1_±5.3_
PIPR	53.0_±2.0_	47.1_±2.4_	56.5_±1.2_	48.3_±0.7_
GNN-PPI	55.1_±1.1_	52.4_±2.1_	59.3_±0.9_	44.8_±3.1_
OntoProtein	56.8_±0.4_	61.2_±1.6_	60.8_±0.8_	48.0_±1.2_
ESM-650M	61.1_±1.0_	**62.9_±1.2_**	63.2_±0.8_	55.2__±0.5__
KEDD (w/o SK)	60.4_±1.5_	55.6_±0.6_	66.8_±1.2_	55.0_±1.2_
KEDD (w/o UK)	62.8_±2.0_	61.3_±1.0_	68.2_±0.9_	55.3_±0.8_
KEDD (w/o SA)	63.4_±1.3_	62.3_±1.2_	68.9_±0.8_	57.2_±0.5_
KEDD	**63.8**_±1.5_	62.7_±1.5_	**69.4**_±1.0_	**57.3**_±1.1_

### Ablation studies

#### Impact of structured and unstructured knowledge

The success of KEDD relies upon the integration of structured and unstructured knowledge, and we explore if these two components contribute equally to each downstream task. We implement two variants of our framework, namely, KEDD (w/o SK) and KEDD (w/o UK), by removing either the structured or unstructured knowledge. The experimental results are presented in Tables S2 and S3 and Tables 2 to 4.

We observe that removing either structured or unstructured knowledge leads to overall performance degradation, indicating that both modalities are indispensable and complementary to each other. Interestingly, structured knowledge plays a more significant role in interaction prediction tasks including DTI, DDI, and PPI. On DP, the impacts of structured and unstructured knowledge vary. For structured knowledge, these results corroborate the proximity hypothesis [51] that if two nodes within the knowledge graph share similar neighbors, they tend to possess analogous properties, connect with the same entity, and share similar embeddings. For unstructured knowledge, we posit that the input texts typically delineate certain aspects of drugs and proteins, which are implicitly connected and occasionally irrelevant to the downstream task. Notably, removing unstructured knowledge leads to a drastic performance decline of 15.9% on Clintox. We posit that the dataset involves predicting the US Food and Drug Administration (FDA) approval state of drugs, which could be described verbatim or inferred from clinical trial outcomes and marketing information within texts.

#### Impact of sparse attention

**Fig. 4.**
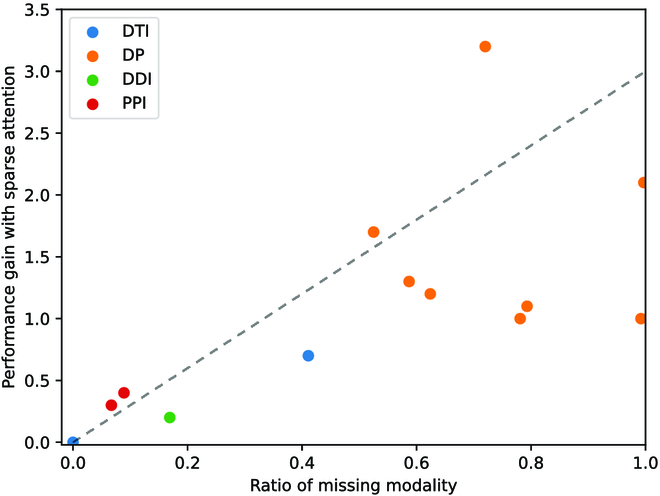
Relationships between performance gain of sparse attention and the ratio of molecules without structured knowledge. Each dot represents the result on a dataset, colored by the corresponding task.

To investigate if the proposed sparse attention mitigates the missing modality problem, we implement KEDD (w/o SA), where we use zero vectors instead of reconstructed features for drugs and proteins that are absent from BMKG. We measure the severity of the missing modality problem by the portion of molecules without structured knowledge, and visualize its relationship with the performance gain attained by sparse attention in Fig. 4. We observe that the benefits of sparse attention are proportional to the severity of the missing modality problem, demonstrating its effectiveness.

#### Impact of modality masking

KEDD proposes modality masking to obtain more training samples for sparse attention and improve robustness. We assess the impact of the masking rate *P* by experimenting on Yamanishi08 dataset under the cold-drug setting. As shown in Table 5, *P* = 0.05 yields the best results. When modality masking is not applied (*P* = 0), the performance deteriorates by 2.4% on average, demonstrating the significance of modality masking. Continued elevation of *P* results in a slight performance decline, suggesting that the reconstructed features may be suboptimal when compared to the original knowledge graph embeddings.

**Table 5.** Performance on DTI using Yamanishi08 dataset under the cold-drug setting with different modality masking probability *P*

*P*	AUROC	AUPR
0.00	78.0_±2.6_	76.4_±2.6_
0.05	**80.4**_**±3.3**_	**78.7**_**±3.8**_
0.10	80.2_±2.5_	78.5_±2.9_
0.20	79.1_±3.0_	77.8_±3.4_

### A case study on real-world drug discovery

**Fig. 5.**
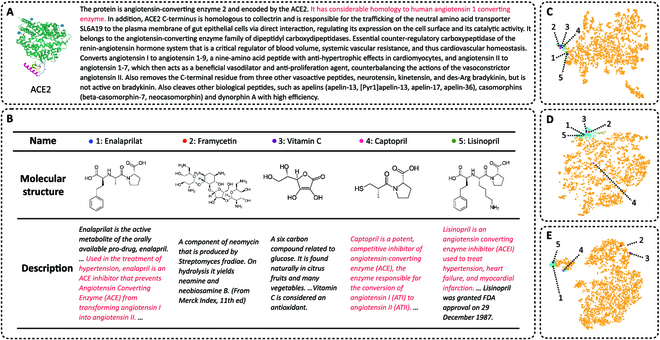
Drug repurposing for ACE2. (A) Details of ACE2. (B) Top five drug candidates, the corresponding molecular structures, and textual descriptions. Expressions related to ACE2 are highlighted. (C) *t*-SNE visualization for molecular features $z_{S}^{D}$. (D) *t*-SNE visualization for structured knowledge features $z_{\mathrm{SK}}^{D}$. (E) *t*-SNE visualization for unstructured knowledge features $z_{\mathrm{UK}}^{D}$. Drugs with >0.5 prediction score based on each modality are highlighted, and the top five drug candidates are marked by different colors and indexes.

To test the power of KEDD in real-world drug discovery scenarios, we perform a case study on drug repurposing involving angiotensin-converting enzyme 2 (ACE2), a protein that has proven to be an entry receptor of severe acute respiratory syndrome coronavirus 2 (SARS-CoV-2) [52,53]. We exclude samples containing ACE2 from the BMKG-DTI dataset and train KEDD on the modified dataset. Then, we predict the probability for each drug within the dataset to interact with ACE2 and select the top five candidates. The heterogeneous inputs of ACE2 and the selected drugs are presented in Fig. 5A and B. To explore the features of each modality, we visualize the features of molecular structure $z_{S}^{D}$, structured knowledge $z_{\mathrm{SK}}^{D}$, and unstructured knowledge $z_{\mathrm{UK}}^{D}$ for each drug via *t*-distributed stochastic neighbor embedding (*t*-SNE) [54] in Fig. 5C to E. More details are presented in Supplementary Section G.

Among the five drugs KEDD identified, captopril and lisinopril are experimentally validated active compounds, whose binding affinity values are reported on PubChem [55]. Additionally, recent studies from the biomedical domain point out that vitamin C and enalaprilat also exhibit lowering effects on the protein [56–58], and an in silico work suggests that framycetin could be a potential ACE2 inhibitor [59].

As shown in Fig. 5C and D, the molecular structure and structured knowledge features for the five drugs are mapped closely to each other, indicating that these modalities tend to play major roles in identifying candidates. Besides, the inhibitory effects of enalaprilat, captopril, and lisinopril on ACE, a homologous protein of ACE2, are pointed out in their text descriptions, and the unstructured knowledge features are within the clusters with high prediction scores solely based on this modality.

From the results, we observe that KEDD is capable of searching potential drugs for novel targets by comprehensively integrating structured and unstructured knowledge. Therefore, there is a possibility for the framework to assist real-world drug discovery applications.

## Discussion

The ability to harness biomedical expertise from diverse multimodal sources holds crucial significance in the realm of biomedical research and drug discovery. KEDD serves as a pioneering work by developing AI models that jointly exploit biomolecular structures, structured knowledge from knowledge bases, and unstructured knowledge from biomedical documents. Remarkably, KEDD can be flexibly applied to wide downstream tasks with minimum modification of model architecture. Besides, we discuss the missing modality problem, a common phenomenon in real-world scenarios where novel drugs and proteins are unrecorded in existing knowledge bases. We present a novel solution by reconstructing feature vectors with sparse attention and modality masking. Through extensive qualitative and quantitative analysis, we validate that both structured knowledge and unstructured knowledge can aid the deficiency of AI models in predicting biomolecular properties and interactions. We also demonstrate the robustness of KEDD when the missing modality problem is pronounced, primarily owing to the proposed sparse attention and modality masking technique. On the drug-repurposing case for ACE2, four of our five prioritized candidates are validated by recent pharmaceutical studies, highlighting the promising potential of our framework in real-world drug discovery.

While KEDD bears promise in accelerating AI drug discovery research, future efforts are expected to address the limitations and further extend the benefits of our framework. First, KEDD predominantly focuses on the acquisition and incorporation of multimodal information, and leverages GraphMVP and MCNN as the drug and protein encoders. More combinations of biomolecular structure modeling approaches, including those that incorporate the 3D geometries of drugs and proteins, could be applied and compared task-by-task to obtain a comprehensive view of different design choices. Second, the application scope of KEDD could be further extended. For example, more biomedical components including diseases, genes, and cellular transcriptomics can also be considered, and more complicated AI drug discovery tasks such as drug–disease interaction prediction [60] and drug response prediction on cell lines [61] can be applied. Finally, the development of interpretable tools is expected to understand how KEDD makes predictions based on molecular structures, knowledge graphs, and biomedical texts. This will also provide more scientific insights for researchers in real-world applications.

## Conclusions

In this work, we present KEDD, an end-to-end deep learning framework for unified AI drug discovery with multimodal knowledge. KEDD builds a novel feature fusion network to jointly harvest the advantages of molecular structure, structured knowledge within knowledge graphs, and unstructured knowledge within biomedical documents. To mitigate the missing modality problem, KEDD leverages sparse attention and a modality masking technique to exploit relevant information from existing knowledge graphs. The effectiveness of KEDD is validated by its state-of-the-art performance on a wide spectrum of downstream tasks, including DTI, DP, DDI, and PPI. With qualitative analysis, we show KEDD’s potential in assisting real-world drug discovery applications.

## Data Availability

The Python code and datasets used in KEDD are https://github.com/icycookies/KEDD_temporal available at https://github.com/PharMolix/OpenBioMed.

## Supplementary Materials

Supplementary 1 Supplementary Sections A to G Figs. S1 to S5 Tables S1 to S3 References [62–77]
