# Supplementary material for: Toward Unified AI Drug Discovery with Multimodal Knowledge
Source: Health Data Sci. 2024 Feb 23;4:0113. doi: 10.34133/hds.0113 (PMC10886071; doi:10.34133/hds.0113)
Supplement: Supplementary 1 — Supplementary Sections A to G Figs. S1 to S5 Tables S1 to S3 References [62–77] [file hds.0113.f1.zip › case_fulltext.pdf]

ACE2	*The protein is angiotensin-converting enzyme 2 and encoded by the ACE2. It has considerable homology to human angiotensin 1 converting enzyme. In addition, ACE2 C-terminus is homologous to collectrin and is responsible for the trafficking of the neutral amino acid transporter SL6A19 to the plasma membrane of gut epithelial cells via direct interaction, regulating its expression on the cell surface and its catalytic activity. It belongs to the angiotensin-converting enzyme family of dipeptidyl carboxydipeptidases. Essential counter-regulatory carboxypeptidase of the renin-angiotensin hormone system that is a critical regulator of blood volume, systemic vascular resistance, and thus cardiovascular homeostasis. Converts angiotensin I to angiotensin 1-9, a nine-amino acid peptide with anti-hypertrophic effects in cardiomyocytes, and angiotensin II to angiotensin 1-7, which then acts as a beneficial vasodilator and anti-proliferation agent, counterbalancing the actions of the vasoconstrictor angiotensin II. Also removes the C-terminal residue from three other vasoactive peptides, neurotensin, kinetensin, and des-Arg bradykinin, but is not active on bradykinin. Also cleaves other biological peptides, such as apelins (apelin-13, [Pyr1]apelin-13, apelin-17, apelin-36), casomorphins (beta-casomorphin-7, neocasomorphin) and dynorphin A with high efficiency.*
Enalaprilat	*Enalaprilat is the active metabolite of the orally available pro-drug, enalapril. Used in the treatment of hypertension, enalapril is an ACE inhibitor that prevents Angiotensin Converting Enzyme (ACE) from transforming angiotensin I into angiotensin II. As angiotensin II is responsible for vasoconstriction and sodium reabsorption in the proximal tubule of the kidney, down-regulation of this protein results in reduced blood pressure and blood fluid volume. Enalaprilat was originally created to overcome the limitations of the first ACE inhibitor, captopril, which had numerous side effects and left a metallic taste in the mouth. Removal of the problematic thiol group from captopril resulted in enalaprilat, which was then modified further with an ester to create the orally available pro-drug enalapril. Enalaprilat is poorly orally available and is therefore only available as an intravenous injection for the treatment of hypertension when oral therapy is not possible.*
Framycetin	*A component of neomycin that is produced by Streptomyces fradiae. On hydrolysis it yields neamine and neobiosamine B. (From Merck Index, 11th ed)*
Vitamin C	*A six carbon compound related to glucose. It is found naturally in citrus fruits and many vegetables. Ascorbic acid is an essential nutrient in human diets, and necessary to maintain connective tissue and bone. Its biologically active form, vitamin C, functions as a reducing agent and coenzyme in several metabolic pathways. Vitamin C is considered an antioxidant.*
Captopril	*Captopril is a potent, competitive inhibitor of angiotensin-converting enzyme (ACE), the enzyme responsible for the conversion of angiotensin I (ATI) to angiotensin II (ATII). ATII regulates blood pressure and is a key component of the renin-angiotensin-aldosterone system (RAAS). Captopril may be used in the treatment of hypertension.*
Lisinopril	*Lisinopril is an angiotensin converting enzyme inhibitor (ACEI) used to treat hypertension, heart failure, and myocardial infarction. Lisinopril and Captopril are the only ACEIs that are not prodrugs. It functions by inhibition of angiotensin converting enzyme as well as the renin angiotensin aldosterone system.ACEIs are commonly used as a first line therapy in the treatment of hypertension, along with thiazide diuretics or beta blockers. Lisinopril was granted FDA approval on 29 December 1987.*
