# Supplementary material for: Toward Unified AI Drug Discovery with Multimodal Knowledge
Source: Health Data Sci. 2024 Feb 23;4:0113. doi: 10.34133/hds.0113 (PMC10886071; doi:10.34133/hds.0113)
Supplement: Supplementary 1 — Supplementary Sections A to G Figs. S1 to S5 Tables S1 to S3 References [62–77] [file hds.0113.f1.zip › Supplementary Materials.docx]

# Supplementary Materials

## A. Curation of the BMKG Dataset

Developing effective and safe drugs requires extensive background knowledge encapsulated either in knowledge bases or biomedical literature. However, such knowledge is scattered among different repositories and publications, which makes access difficult for computational methods. To this end, we constructed BMKG with 6,917 drugs, 19,992 proteins, and 2,905,076 relationships between them. BMKG provides comprehensive information for biomolecules, including their molecular structures (SMILES strings for drugs and amino acid sequences for proteins), interaction relationships, and text descriptions. Figure S1 gives a brief illustration of how the dataset is constructed. We first obtain compounds and proteins commonly used in drug discovery from multiple primary sources, as shown in Figure S1(a). Then, we extract and standardize the relationships and additional information of the selected molecules from multiple additional repositories, as shown in S1(b), to enrich the BMKG knowledge graph illustrated in Figure S1(c).

Figure S1. The construction process of BMKG. a We collect information from primary sources including DrugBank and UniProt. Both databases consist of expert-written descriptions for drugs and proteins. b We unify the format of the SMILES strings for molecules with RDKit. We connect drugs and proteins using intermediate databases including Entrenz Gene and HGNC. We obtain protein-protein interactions from the human PPI network, BioGRID, and STRING. c We obtain BMKG, a large-scale dataset with biomolecular structures, interaction relationships, and textual descriptions.

**Step 1. Extracting drugs and proteins with supplementary information from primary sources.** For molecules, we select DrugBank v5.1.8 [30], a popular database containing comprehensive information about drugs, as the primary data source. DrugBank contains 7,803 unique drugs in total. By removing drugs without SMILES strings, we obtain 6,917 drugs, and 3,625 of them are provided with expert-written textual descriptions. Then, we transform the XML file in DrugBank into a dictionary where each key corresponds to the DrugBank ID and the value includes the text description and SMILES string, which is unified by RDKit [62]. For proteins, we use the widely-adopted database UniProt Swiss-Prot3 [33], which provides 19,992 proteins with amino acid sequences, and 16,591 of them have descriptive texts. Similarly, we build a dictionary to map UniProt IDs to the corresponding protein sequences and texts.

**Step 2. Building the interaction network.** Based on DrugBank, we extract 2,223,850 synergistic relationships between drugs. It also provides a total of 47,530 drug targets, drug enzymes, drug carriers, and drug transporters. These drug receptors could be found in Entrez Gene [63], a resource maintained by NCBI that contains a large scale of gene-specific data. To obtain the correspondence between Entrez Gene and UniProt Swiss-Prot, we use HUGO Gene Nomenclature Committee (HGNC)as an intermediary [64]. Finally, we obtain 42,961 edges connecting drugs and proteins.

For protein-protein interactions, we combine the human PPI network [65], BioGRID [66], and the STRING database [67]. The processed data contains 633,696 protein-protein interactions. It includes 10 types of interactions, e.g. regulation, metabolic pathways, kinase-substrate and protein complex interactions.

## B. Evaluation Datasets and Splitting Protocols

### 1. Drug-Target Interaction Prediction (DTI)

We incorporate Yamanishi08’s dataset and the BMKG-DTI dataset for this task.

- Yamanishi08’s dataset consists of four subsets for the interacting enzymes, ion channels, G-protein-coupled receptors (GPCR), and nuclear receptors (NR). We combine the four subsets as positive samples and incorporate the same randomly-picked negative samples as in KGE_NFM [13]. The dataset encompasses 791 drugs, 989 proteins, 5,127 positive samples, and 5,127 negative samples.
- The BMKG-DTI is curated based on the drug-protein interactions within the BMKG dataset (more details in Supplementary Section C). It consists of 2,803 drugs, 5,417 proteins, 19,280 positive samples, and 28,211 negative samples. BMKG-DTI is devoid of the missing modality problem, which could demonstrate the full potential of KEDD by incorporating molecular structures, structured knowledge and unstructured knowledge.

We split the two datasets into 5 folds for the warm-start, cold-drug, and cold-protein settings, and 9 folds for the cold-cluster setting. The details of our splitting protocols are as follows:

- In the warm-start setting, we randomly partition data samples into 5 different folds. In this setting, drugs and proteins within the test set are most witnessed during training.
- In the cold-drug setting, we randomly split the drugs into 5 sets of equal size. Each fold consists of drug-target interactions exclusively sourced from a single set of drugs. In this setting, drugs within the test set are unseen during training.
- In the cold-protein setting, we randomly split the proteins into 5 sets of equal size. Each fold consists of drug-target interactions exclusively sourced from a single set of proteins. In this setting, proteins within the test set are unseen during training.
- In the cold-cluster setting, we measure the similarity between drugs by the Jaccard Distance between RDKit molecular fingerprints, and the similarity between proteins by the cosine similarity between normalized CTD descriptors. Then, we apply single-linkage clustering [68] to split drugs and proteins into 3 clusters based on the calculated similarity. The drug-target interactions are partitioned into a 33 grid by the clusters. For each fold, we select one cluster of drugsand one cluster of proteins, and the test set corresponds to one grid. Then, data samples within the same column or the same row as the grid are withdrawn, and the remaining 4 grids are picked as the training set. In this way, we ensure that both drugs and proteins within the test set are disjoint and structurally different from the training set.

### 2. Drug Property Prediction (DP)

We adopt 8 popular benchmarks from MoleculeNet, detailed as follows:

- BBBP aims to predict if a drug could penetrate the blood-brain barrier. It contains 2,053 drugs.
- ClinTox aims to predict the clinical trial toxicity and the FDA approval status for 1,491 drugs.
- Tox21 consists of toxicity information on 12 targets over 7,831 drug compounds.
- SIDER aims to predict the side effects of 1,427 drugs on 27 classes of organs.
- ToxCast aims to predict bioassay outcomes on 617 tasks including nuclear receptors and stress response pathways for 8,575 drugs.
- MUV aims to predict 17 different bioassay outcomes sampled from PubChem. It consists of a total of 93,087 drugs.
- HIV aims to predict if a drug exhibits the ability to inhibit HIV replication. It is composed of 41,127 drugs.
- BACE aims to predict the binding results of 1,513 drugs for a set of inhibitors of human -secretase 1 (BACE-1).

The datasets are partitioned by Scaffold split. Specifically, molecules with similar scaffolds are assigned to the same cluster, and the clusters are further split into train, validation, and test sets with a ratio of 8:1:1. This is a more challenging scheme that is closer to real-world scenarios, wherein molecules within the test set are structurally different from those in the training set.

### 3. Drug-Drug Interaction Prediction (DDI)

For this task, we adopt Luo’s dataset. The original dataset consists of heterogeneous information including drug-drug interactions, drug-disease relationships, and drug-side effect relationships. We sample the drug-drug interaction subset with 721 drugs and 494,551 possible interactions. We adopt the warm-start split, where data samples are randomly partitioned into 10 folds.

### 4. Protein-Protein Interaction Prediction (PPI)

We adopt the SHS27k and SHS148k datasets, detailed as follows:

- SHS27k is randomly sampled from the homo sapiens subset of the STRING database, which divides PPI into 7 types, namely reaction, binding, post-translational modifications (ptmod), activation, inhibition, catalysis, and expression. SHS27k contains 1,690 proteins with sequence identity and 7,624 samples.
- SHS148k is also sampled from the homo sapiens subset of the STRING database. Similarly, it consists of 5,189 proteins with sequence identity and 44,488 samples.

Following GNN_PPI [18], we incorporate the DFS (depth-first search) and BFS (breadth-first search) splits that simulate real-world cold-start scenarios.

- The DFS split randomly selects a protein as the root node if there exists less than *t* proteins that interact with it. Then, it applies the depth-first search by iteratively moving to one of the neighbors of the current node. The proteins that have been traversed, which are sparsely distributed and connected with each other, are assigned to the test set. PPIs that connect proteins within the test set are incorporated, and the iteration is terminated if the test set contains over 20%PPIs.

- Figure S2. Construction process of the BMKG-DTI dataset.
- The BFS split follows the same selection process of the root node as DFS. Then, it applies the breadth-first search by traversing every neighbor of the current node and assigning them to the test set before moving to the next node. The proteins within the test set are densely connected and form clusters. Similarly, PPIs that connect proteins within the test set are incorporated, and the iteration is terminated if the test set contains over 20% PPIs.

## C. Curation of BMKG-DTI Dataset

The number of drugs and proteins with additional knowledge inputs in existing DTI datasets is not satisfying, which restricts the full power of KEDD. Thus, based on the BMKG dataset we constructed, we further build a DTI dataset BMKG-DTI. The dataset aimsto predict the binding relationship between 2,803 drugs and 2,810 proteins. It contains 19,280 binding pairs and 28,211 non-binding pairs. The construction process, shown in Figure S2, is composed of two steps:

**Step 1. Selecting appropriate drugs, proteins, and drug-target interactions from BMKG.** We extract 2,803 drugs and 5,417 proteins with at least one drug-protein edge in BMKG. Then, we randomly sample 2,810 proteins to balance the number of compounds and proteins.

**Step 2. Generating negative drug-protein pairs that are most unlikely to bind with.** Generating appropriate negative samples is a challenging problem widely discussed in previous studies [69,70], from which we address two major concerns. First, the sampled drug-protein pair should exhibit minimal potential to interact with each other. In addition, the ratio between positive pairs and negative pairs for each drug and protein should be similar to avoid hidden bias. To address these problems, we propose a negative sampling pipeline shown in Figure S2(c). For each drug-protein positive pair , where *d*, *p*, and denote drug, protein, and the label, we fix the drug extitd to select a non-interacting protein and fix the protein *p* to select a non-interacting drug , thus formulating two negative samples and . When the drug is fixed, we first obtain the 1-hop and 2-hop neighbors of *p* by breadth-first search and sample 100 proteins from the remaining nodes. If the number of remaining proteins is less than 100, all of them are selected. We deem that *p* and are unlikely to be similar and interact with the same compound if they are far from each other in a knowledge graph. Then, we rank these proteins based on their structural similarity with *p* and randomly select from the top-3 results with minimum similarity. The similarity is measured by the Smith-Waterman Score [71], which is a widely adopted protocol for sequence proximity. When the protein is fixed, we use the same process to select the non-interacting drug , except that the similarity is calculated by the Jaccard score of the 1024-dimensional RDKit molecular fingerprint. Finally, we remove duplicate drug-protein pairs and obtain 28,211 negative samples.

## D. Details for KEDD

### 1. Illustration for KEDD in Drug Property Prediction


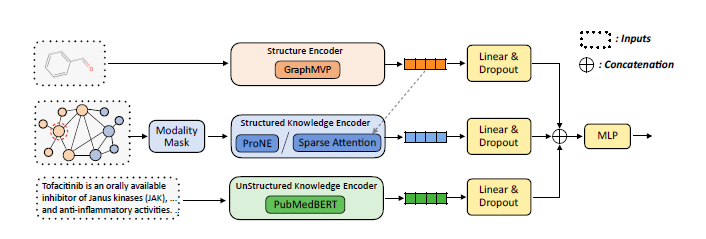


Figure S3. The KEDD framework for DP.

Different from interaction-prediction tasks, the inputs of drug property prediction only consist of one molecule, and the branch for the other molecule becomes empty. We visualize the KEDD architecture in Supplementary Figure S3 for clarity.

### 2. More Implementation Details for KEDD

Table S1. Details of hyperparameters.

| **Name** | **Descriptions** | **Search Space** |
| --- | --- | --- |
|  | Hidden dimensions of structure features |  |
|  | Hidden dimensions of SK features |  |
|  | Hidden dimensions of UK features |  |
|  | Dropout ratio for SK features |  |
|  | Dropout ratio for UK features |  |
| lr | Learning rate |  |
| batch_size | Batch size |  |
| epochs | Number of training epochs |  |

The hyperparameters for KEDD are presented in Supplementary Table S1. KEDD is implemented using PyTorch, pytorch geometrics, RDKit, transformers, and scanpy.

## E. Details for Baseline Models

### 1. Baselines for DTI

For this task, we incorporate machine learning models including random forest (RF) [41] and support vector machine (SVM) [42]. We concatenate the 1,024-dimensional molecule fingerprint for drugs and the 128-dimensional CTD descriptor [72] for proteins as input features for the two models.

We also incorporate structure-based deep learning models including:

- DeepDTA [43] encodes each character of the SMILES string with one-hot encoding, and incorporates 3 layers of convolutional networks to encode the drug structure. Similarly, each amino acid of the protein sequence is transformed into the one-hot encoding and fed into 3 convolutional layers to calculate the protein features. Then, the two features are concatenated and passed into a multi-layer perceptron for DTI prediction.
- GraphDTA [44] incorporates graph neural networks to obtain drug features based on its 2D molecular graph. Since the molecular structure encoder of KEDD is built upon the GIN network [25], we evaluate the model using GIN as the backbone. The protein encoder is also a 3-layer convolution network. Similar to DeepDTA, GraphDTA makes predictions by concatenating drug and protein features, and feeding them into an MLP.
- MGraphDTA [26] is a powerful model specially designed for the DTI task. It incorporates a super-deep GNN architecture to capture the global features of the 2D molecular graphs. The protein encoder is the same as our model, which is a multi-scale convolution network with three branches of convolution networks consisting of 1, 2, and 3 layers respectively. The prediction block follows the same as DeepDTA and GraphDTA.

Additionally, we incorporate a multimodal approach:

- KGE_NFM [13] first calculates knowledge graph embeddings with DistMult [73]. Then it performs dimension reduction with PCA. It concatenates the knowledge graph embeddings for drugs and proteins, as well as the molecular fingerprints and protein descriptors to formulate the overall feature. Then, it leverages the NFM model [74] to make predictions.

### 2. Baselines for DP

To the best of our knowledge, multimodal approaches are rarely tapped in predicting drug properties. Hence, we incorporate unimodal baselines that model molecular structures. For machine learning models including RF and SVM, we incorporate the 1,024-dimensional molecule fingerprint as inputs. For deep learning models, we evaluate:

- GIN [25] follows the same 5-layer graph neural architecture as KEDD’s molecule encoder. Its parameters are initialized randomly without pre-training.
- MolCLR [9] is composed of 5 GIN layers to encode the molecular graph. The model is pre-trained on large-scale molecules by contrastive learning between different views of the same molecule.
- KV-PLM [10] is a Transformer-based model that adopts the BERT architecture [75] to encode the SMILES strings of molecules. It is pre-trained on both SMILES strings and drug-related texts with a mask language model(MLM) objective.
- MoMu [45] also adopts the 5-layer GIN architecture. However, it incorporates multimodal information by pre-training with a contrastive objective between the molecular structures and corresponding textual descriptions.
- MoCL [46] follows the same architecture as MolCLR. The difference between the two models lies in the pre-training stage, where MoCL proposes to treat structurally similar molecules as positive samples.
- GraphMVP [8] is also a 5-layer GIN pre-trained by contrastive learning between the representations of 2D topologies and 3D geometries.

### 3. Baselines for DDI

Machine learning baselines include RF and SVM. Similarly, we concatenate the molecule fingerprint of two drugs as input features.

The unimodal baselines that incorporate structured knowledge from biomedical knowledge graphs include:

- DeepDTnet [47] is a network-based that embeds a heterogeneous knowledge graph into vector representations with deep neural networks (DNNs). The likelihood of two drugs to interact with each other is calculated by the dot productof their node representations.
- DTINet [38] is another knowledge graph mining model that encodes the network topology using the random walk with restart algorithm.
- DeepR2cov [48] leverages a bidirectional Transformer pre-trained on 3 billion meta-paths within a heterogeneous knowledge graph to encode node embeddings for drugs. Then, it uses an IMC model [76] to predict DDIs.
- MSSL2drug [49] performs multi-task self-supervised pre-training on biological networks to calculate node representations of two drugs, and feed them into an MLP for DDI prediction.

The multimodal baselines that incorporate both molecular structures and structured knowledge include:

- DDIMDL [50] designates 4 independent DNNs to model the similarities between the chemical substructures, drug-target interactions, drug-enzyme interactions, and drug-pathway relationships of two drugs. The outputs of the DNNsare concatenated and fed into an MLP to predict DDIs.
- KGE_NFM [13] is initially designed to predict drug-target interactions. In DDI prediction, we concatenate the knowledge graph embeddings and molecular fingerprints of two drugs and feed the results into the NFM model.

### 4. Baselines for PPI

For PPI, the inputs for RF and SVM are the concatenation of the CTD descriptors for two proteins.

The unimodal baselines that mine protein structures include:

- PIPR [39] proposes a Siamese residual RCNN architecture to model PPIs. It comprises several RCNN blocks, each of which is composed of a convolution layer, a pooling layer, and a bidirectional GRU with residual shortcuts. The sequence features of the two proteins are concatenated and fed into an MLP to predict PPIs.
- ESM-650M [11] is a large protein language model with 33 Transformer layers and a total of 650 million parameters. The model is pre-trained on billions of protein sequences with an MLM objective. We perform max-pooling over the output features of the last layer to obtain protein features. Then, we concatenate the features of two proteins and train a 2-layer MLP to predict PPIs.

The multimodal baselines that incorporate protein-related structured knowledge and protein structures include:

- GNN-PPI [18] encodes protein sequences with one layer of RCNN in PIPR. The node embeddings of the PPI network are initialized by the structural features. Then, the node features are propagated within multiple layers of GIN. The existence of a PPI is identified by feeding the dot product of two node features into a fully connected layer.
- OntoProtein [19] incorporates ProtBERT to encode protein sequences and initialize knowledge graph embeddings. Then, it leverages a TransE objective to incorporate the knowledge within relationships between proteins and gene ontologies into the protein encoder. The PPI prediction module is the same as ESM-650M.

## F. Supplementary Experiment Tables

911

Table S2. Experiment results on the Yamanishi08’s dataset. w/o SK: without structured knowledge; w/o UK: without unstructured knowledge; w/o SA: without sparse attention.

| Model | **Warm** | | **Cold Drug** | | **Cold Protein** | | **Cold Cluster** | |
| --- | --- | --- | --- | --- | --- | --- | --- | --- |
|  | AUROC | AUPR | AUROC | AUPR | AUROC | AUPR | AUROC | AUPR |
| RF |  |  |  |  |  |  |  |  |
| SVM |  |  |  |  |  |  |  |  |
| DeepDTA |  |  |  |  |  |  |  |  |
| GraphDTA |  |  |  |  |  |  |  |  |
| MGraphDTA |  |  |  |  |  |  |  |  |
| KGE_NFM |  |  |  |  |  |  |  |  |
| KEDD (w/o SK) |  |  |  |  |  |  |  |  |
| KEDD (w/o UK) |  |  |  |  |  |  |  |  |
| KEDD (w/o SA) |  |  | **80.7** | **80.2** |  | **88.5** |  |  |
| KEDD | **96.6** | **96.4** |  |  | **88.5** | **88.5** | **74.7** | **73.9** |

Table S3. Experiment results on the BMKG-DTI dataset. w/o SK: without structured knowledge; w/o UK: without unstructured knowledge. Given the absence of the missing modality problem in this dataset, we omit reporting the results of KEDD when the sparse attention module is removed, as it does not exert any impact.

911

| Model | **Warm** | | **Cold Drug** | | **Cold Protein** | | **Cold Cluster** | |
| --- | --- | --- | --- | --- | --- | --- | --- | --- |
|  | AUROC | AUPR | AUROC | AUPR | AUROC | AUPR | AUROC | AUPR |
| RF |  |  |  |  |  |  |  |  |
| SVM |  |  |  |  |  |  |  |  |
| DeepDTA |  |  |  |  |  |  |  |  |
| GraphDTA |  |  |  |  |  |  |  |  |
| MGraphDTA |  |  |  |  |  |  |  |  |
| KGE_NFM |  |  |  |  |  |  |  |  |
| KEDD (w/o SK) |  |  |  |  |  |  |  |  |
| KEDD (w/o UK) |  |  |  |  |  |  |  |  |
| KEDD | **96.6** | **95.5** | **92.5** | **90.4** | **93.7** | **91.9** | **88.4** | **85.4** |

## G. Details for the Case Study


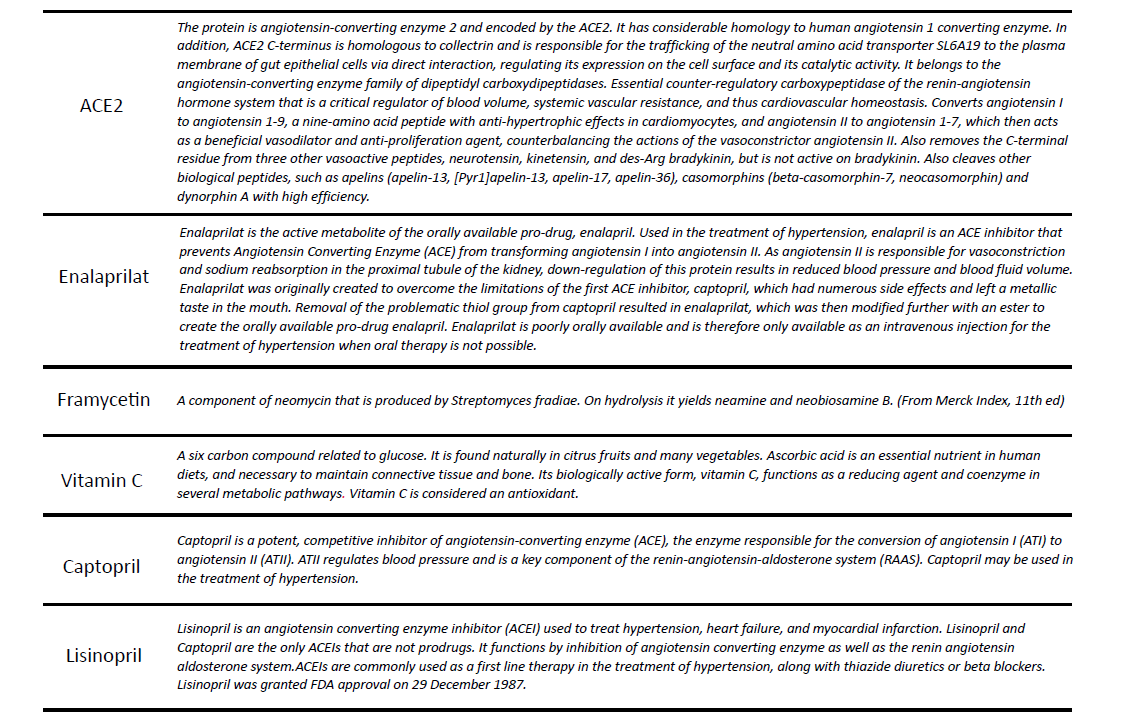


Figure S4. Full description texts for ACE2 and drug candidates in the case study


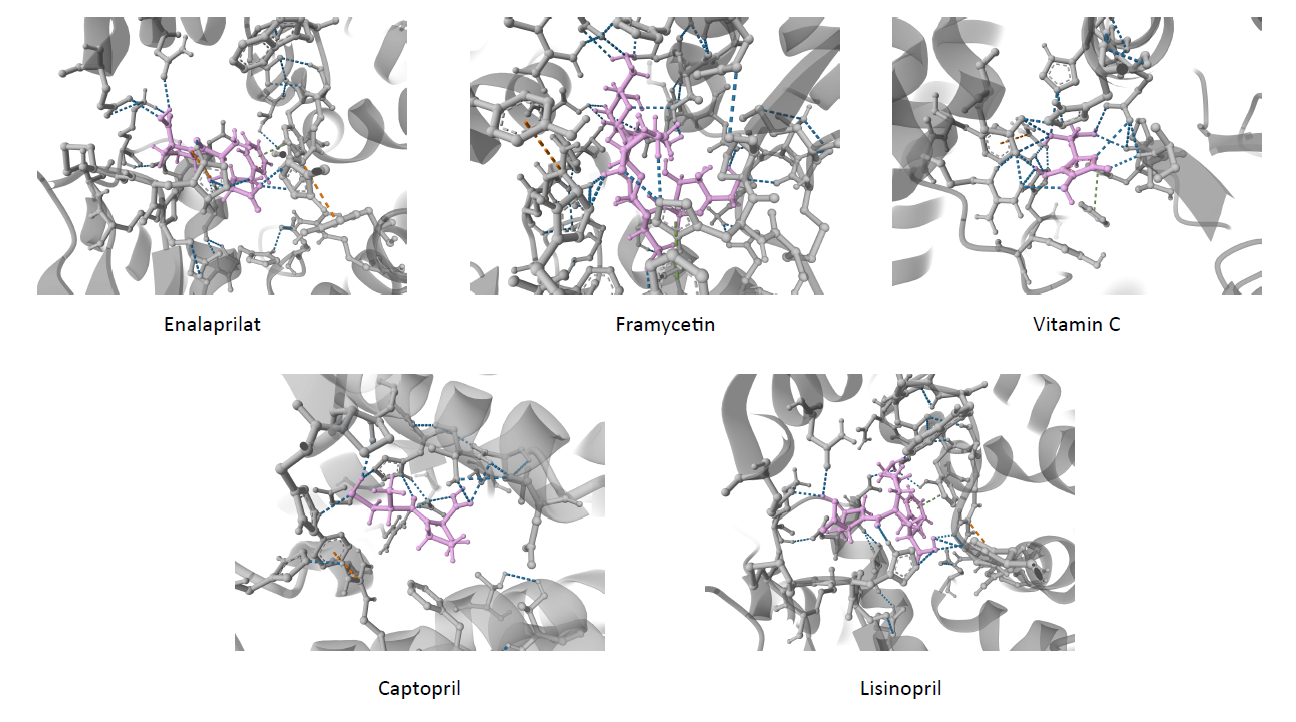
Figure S5. The molecular docking results between ACE2 and the 5 drug candidates. The protein target and the drug ligand are colored by grey and plum, respectively. Hydrogen bonds are visualized by blue dashed lines, and cation-Pi interactions are visualized by orange dashed lines.

Detailed textual descriptions for ACE2 and the 5 drug candidates are presented in Supplementary Figure S4. Additionally, we perform molecular docking between the protein target and the selected drug ligands with the AutoDock Vina [77] software. The docking results are illustrated in Supplementary Figure S5.
