# Supplementary figures and images for: Toward Unified AI Drug Discovery with Multimodal Knowledge
Source: Health Data Sci. 2024 Feb 23;4:0113. doi: 10.34133/hds.0113 (PMC10886071; doi:10.34133/hds.0113)

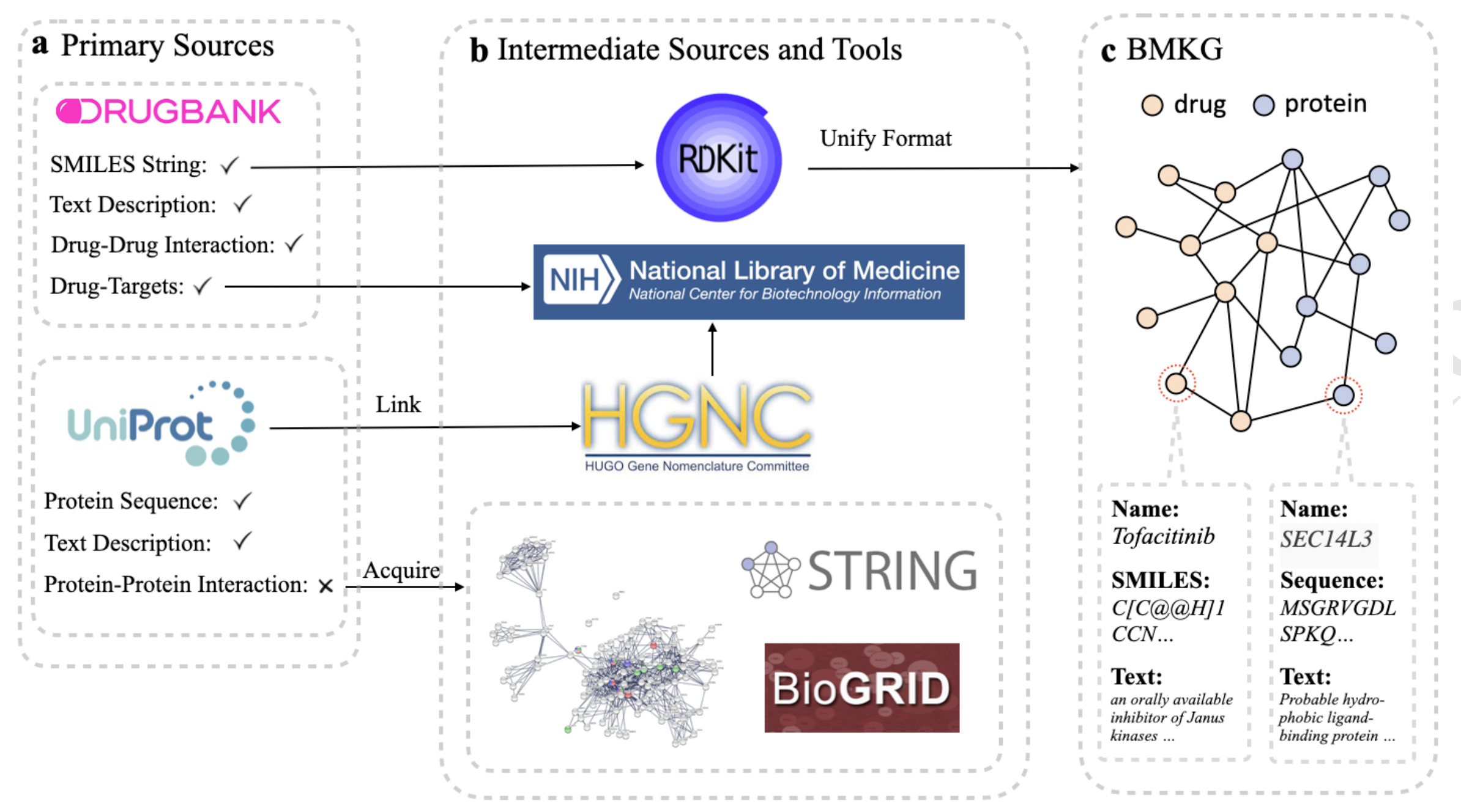

Supplement: Supplementary 1 — Supplementary Sections A to G Figs. S1 to S5 Tables S1 to S3 References [62–77] [file hds.0113.f1.zip › bmkg.png]

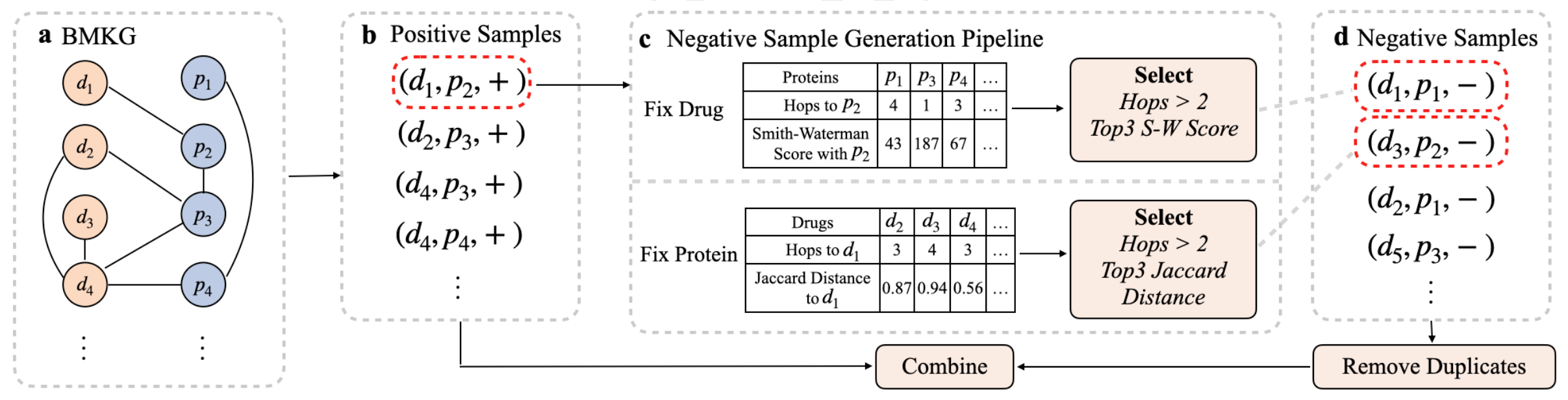

Supplement: Supplementary 1 — Supplementary Sections A to G Figs. S1 to S5 Tables S1 to S3 References [62–77] [file hds.0113.f1.zip › bmkg-dti.png]

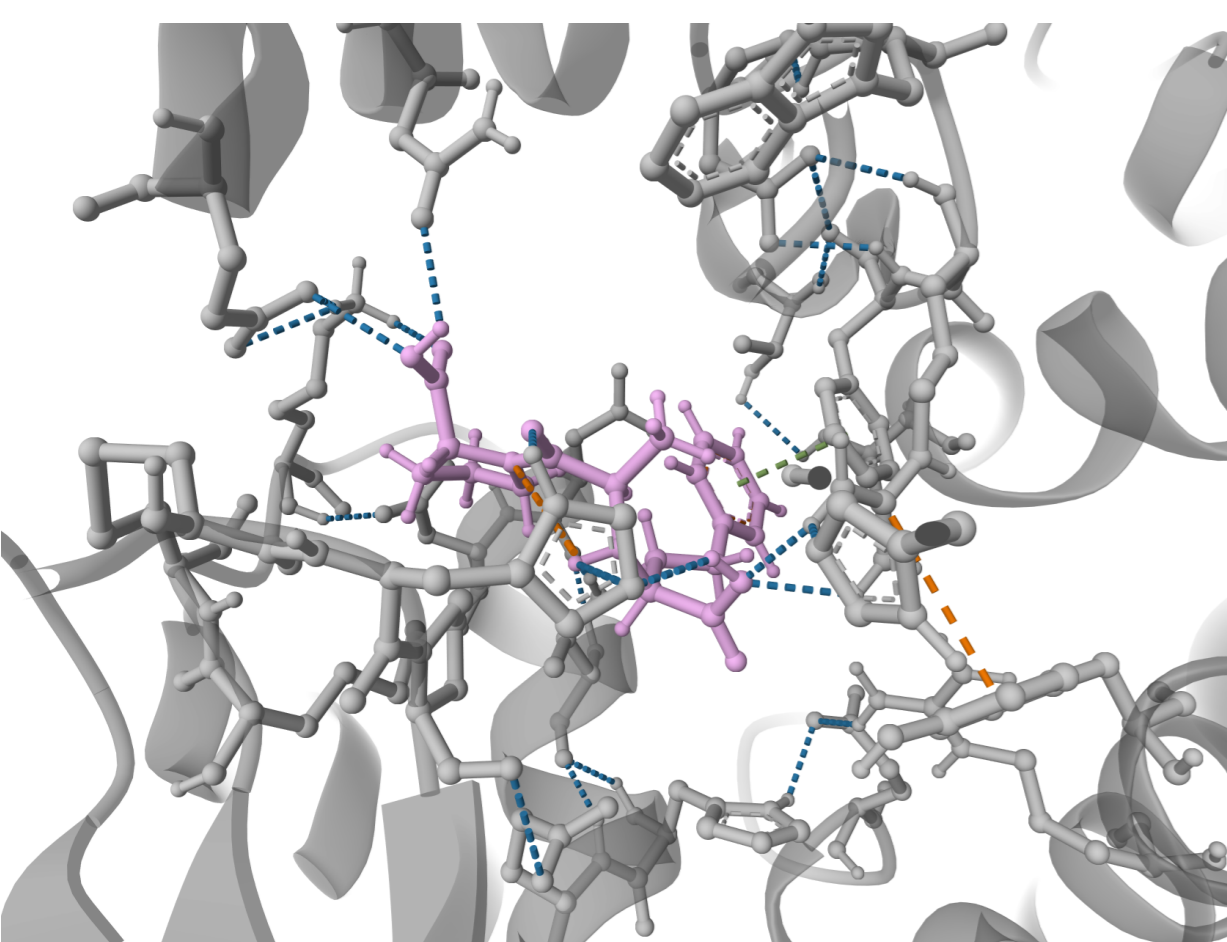

Enalaprilat

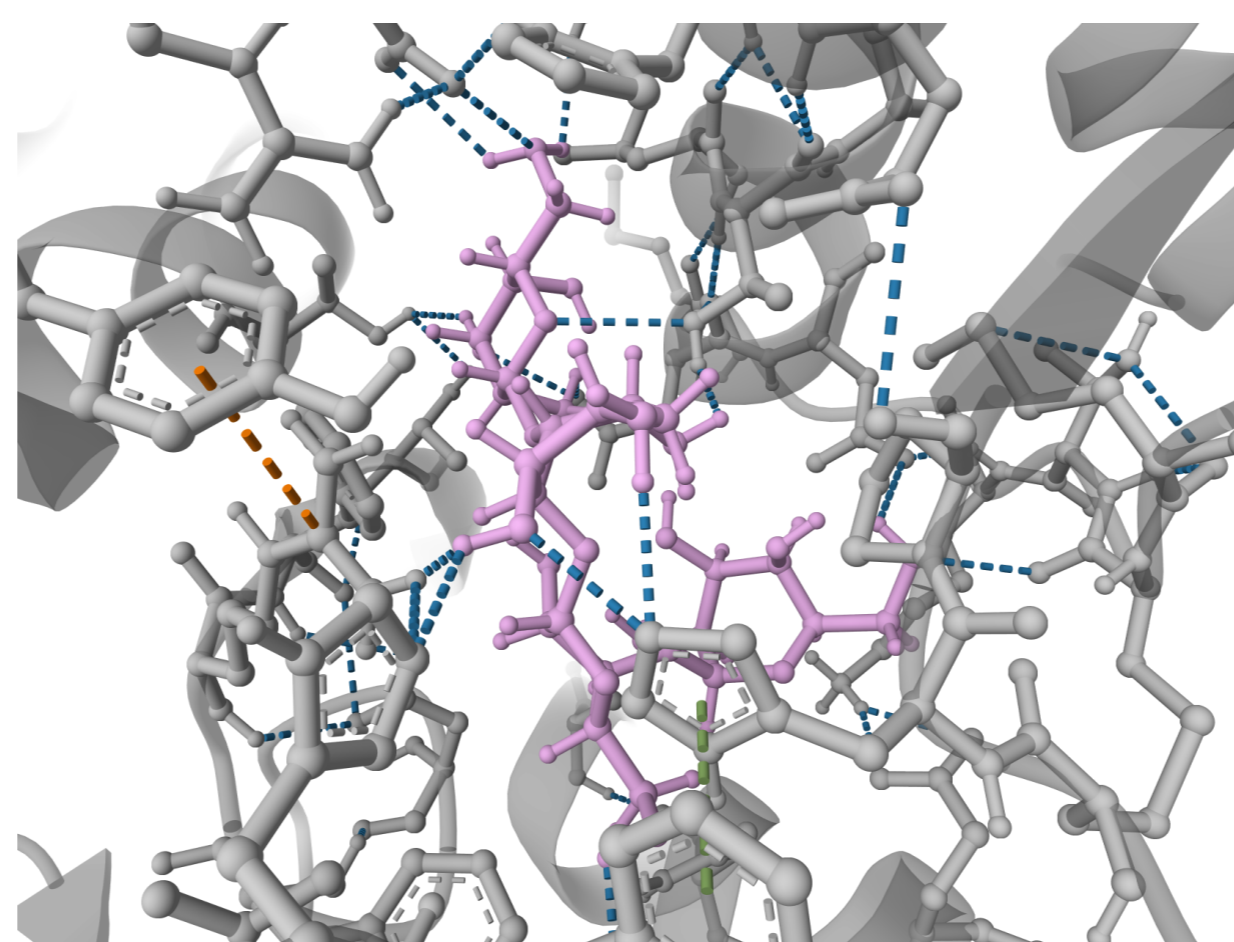

Framycetin

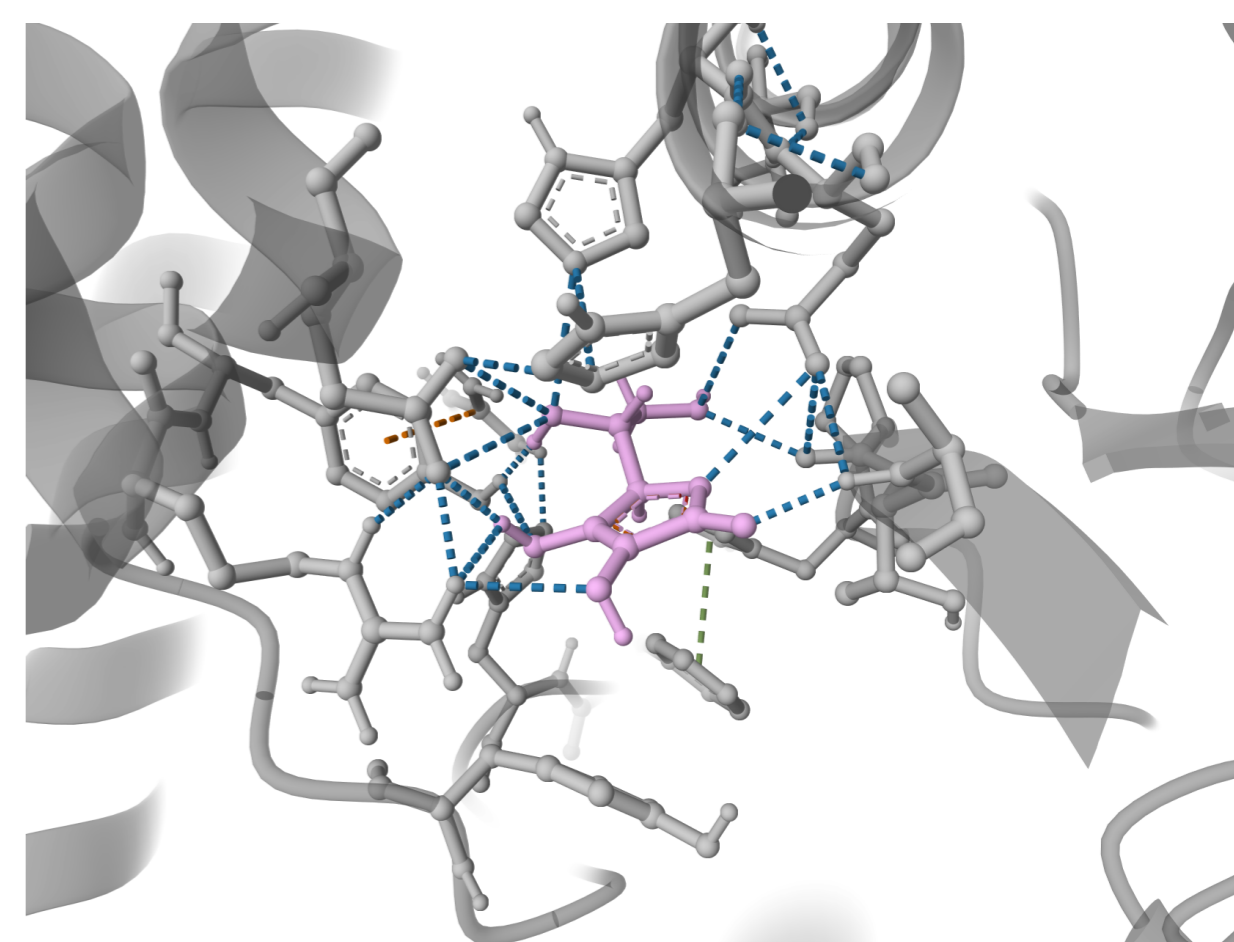

Vitamin C

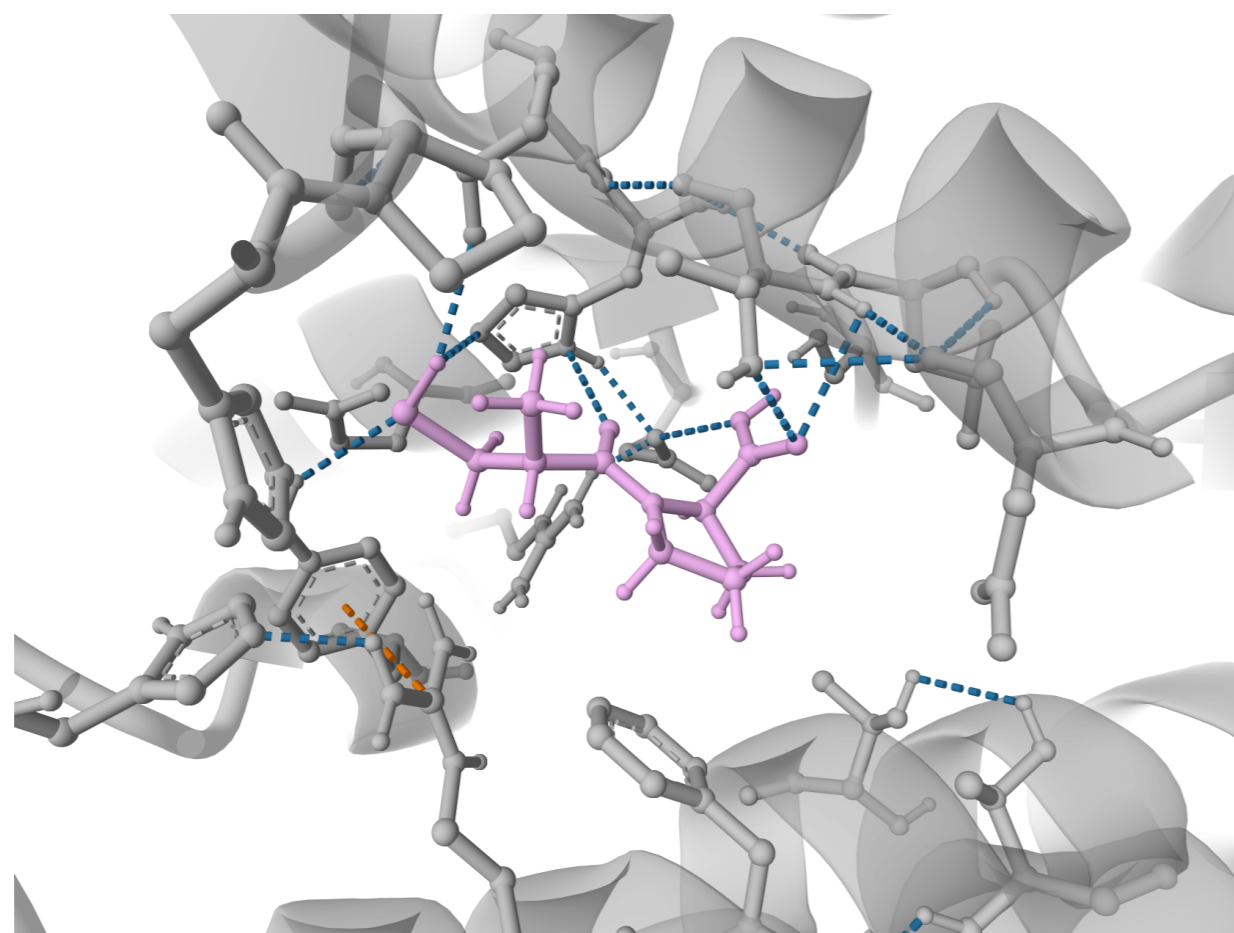

Captopril

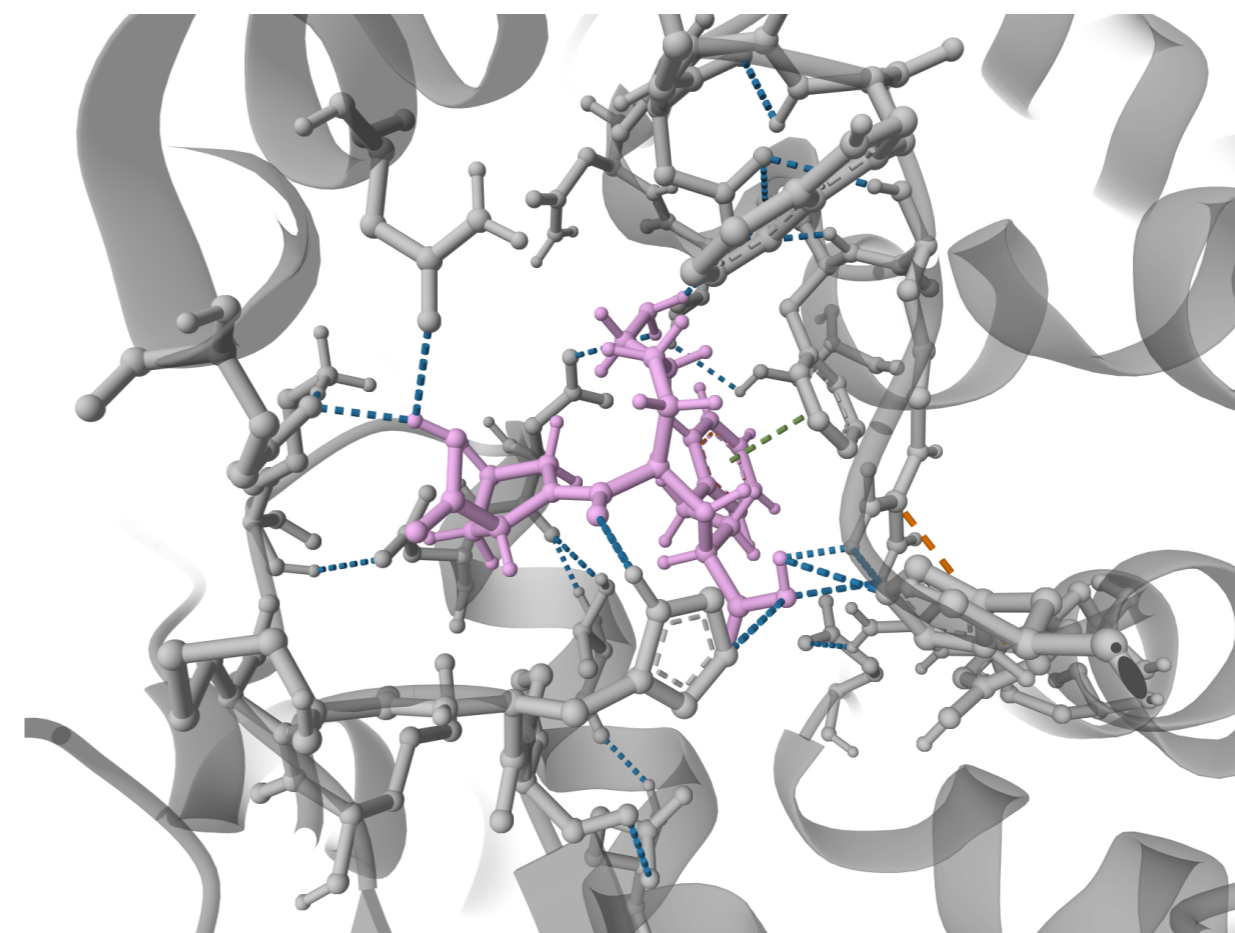

Lisinopril

Supplement: Supplementary 1 — Supplementary Sections A to G Figs. S1 to S5 Tables S1 to S3 References [62–77] [file hds.0113.f1.zip › case_docking.pdf]

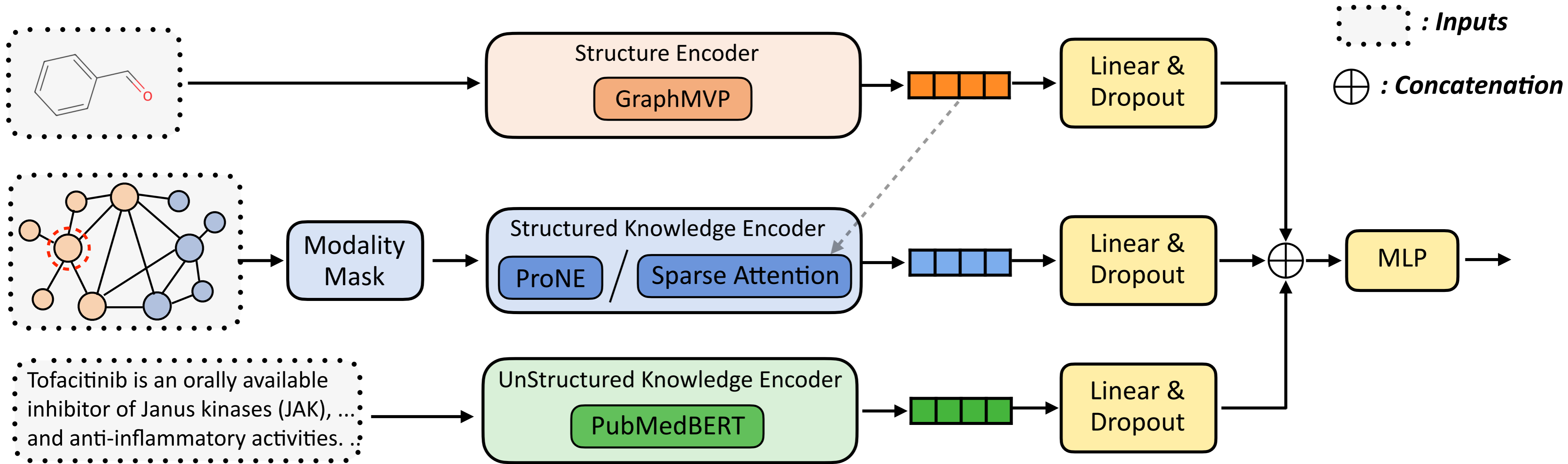

Supplement: Supplementary 1 — Supplementary Sections A to G Figs. S1 to S5 Tables S1 to S3 References [62–77] [file hds.0113.f1.zip › model_dp.pdf]
